# Efficacy and safety of oral Chinese patent medicine combined with quadruple therapy for chronic atrophic gastritis: a systematic review and network meta-analysis

**DOI:** 10.3389/fmed.2026.1859753

**Published:** 2026-06-23

**Authors:** Dan Han, Yang Yu, Bo-yi Wang, Zhan-ze Ma

**Affiliations:** 1 Department of Integrated Traditional Chinese and Western Medicine, Liaoning University of Traditional Chinese Medicine Xinglin College, Shenyang, China; 2 Shenyang Second Traditional Chinese Medicine Hospital, Shenyang, China; 3 Organization Department, Liaoning University of Traditional Chinese Medicine, Shenyang, China

**Keywords:** chronic atrophic gastritis, gastrointestinal hormone, inflammatory factor, network meta-analysis, oral Chinese patent medicines, pepsinogen, quadruple

## Abstract

**Background:**

Chronic atrophic gastritis (CAG) is an important precancerous lesion of gastric cancer, with *Helicobacter pylori* (*H. pylori*) infection being a major etiological factor. Although quadruple therapy is the first-line treatment for *H. pylori* eradication, it still has certain limitations. The comparative efficacy of oral Chinese patent medicines combined with quadruple therapy remains unclear. Therefore, this study conducted a network meta-analysis (NMA) to systematically evaluate their efficacy and safety.

**Methods:**

Randomized controlled trials (RCTs) on oral Chinese patent medicines combined with quadruple therapy for chronic atrophic gastritis were searched electronically in databases including China National Knowledge Infrastructure (CNKI), VIP, WanFang, Chinese Biomedical Literature Database (CBM), PubMed, Web of Science, and Cochrane Library from January 1, 2016, to April 1, 2026. Network Meta-analysis was performed on the included RCTs using Stata and R software.

**Results:**

A total of 45 RCTs were included, involving 4,465 participants and 10 kinds of oral Chinese patent medicines. The network Meta-analysis showed that the 10 oral Chinese patent medicines (OCPMs) combined with quadruple therapy (QT) could effectively eradicate *H. pylori* eradication rates, improve gastrin and pepsinogen levels, and reduce inflammatory factor levels. The SUCRA ranking indicated that, Weisu granule combined with QT may be associated with greater benefit for improving *H. pylori* eradication rate, increasing motilin levels, and reducing interleukin-6 and tumor necrosis factor-*α* levels. Weifuchun tablets showed a favorable SUCRA ranking for increasing gastrin levels and reducing C-reactive protein and interleukin-8 levels. Moluo pills showed the highest SUCRA ranking for reducing interleukin-1β levels, while Jinghua Weikang Capsule showed the highest SUCRA probability for improving pepsinogen I levels. In addition, Biling Weitong granule ranked highest by SUCRA for improving pepsinogen II levels and the pepsinogen I/pepsinogen II ratio. However, these findings are based on indirect evidence and require confirmation in future head-to-head trials.

**Conclusion:**

Within the limitations of low-certainty evidence, the 10 oral Chinese patent medicines combined with quadruple therapy showed exploratory trends in improving *H. pylori* eradication rates, gastrin, pepsinogen, and inflammatory factor levels in patients with chronic atrophic gastritis, with few adverse reactions. However, the evidence network was predominantly star-shaped without closed loops, lacking direct head-to-head comparisons among different patent medicines. Restricted by the small sample size, methodological flaws and high clinical heterogeneity of included trials, the current findings should be regarded as hypothesis-generating rather than definitive clinical conclusions. Further large-sample, multicenter, high-quality randomized controlled trials are still required to verify our exploratory results and support precise clinical decision-making.

**Systematic review registration:**

https://www.crd.york.ac.uk/PROSPERO/view/CRD420261366962, CRD420261366962.

## 1 Introduction

Chronic atrophic gastritis (CAG) is a chronic inflammatory disease characterized by the reduction or loss of gastric mucosal glands, with or without intestinal metaplasia and dysplasia, and is widely recognized as an important precancerous lesion in the development of gastric cancer (1). According to the Correa cascade model, the gastric mucosa may undergo a sequential progression from chronic superficial gastritis to atrophic gastritis, intestinal metaplasia, intraepithelial neoplasia, and ultimately gastric cancer (2). Therefore, early identification and effective intervention of CAG are of great significance for preventing disease progression and reducing the risk of gastric cancer.

CAG has a relatively high prevalence worldwide, particularly in East Asia (such as China, Japan, and South Korea), which is closely associated with a higher prevalence of *Helicobacter pylori* (*H. pylori*) infection and dietary patterns (3). Epidemiological surveys in China indicate that the prevalence of CAG increases significantly with age, with middle-aged and elderly populations being the main affected groups. In addition, long-term unhealthy dietary habits, such as high salt intake and consumption of pickled foods, as well as smoking, alcohol consumption, and chronic *H. pylori* infection, are considered important risk factors (4). Due to its insidious onset and lack of specific symptoms, CAG is often detected during routine physical examinations or endoscopic evaluations, further increasing the complexity of disease management.

*H. pylori* infection is considered one of the key etiological factors in the occurrence and progression of CAG. Current clinical guidelines recommend a quadruple therapy (QT) consisting of a proton pump inhibitor (PPI), two antibiotics, and bismuth as the first-line regimen for *H. pylori* eradication (5). Although this regimen has certain efficacy in eliminating *H. pylori*, it still has notable limitations. On the one hand, with the increasing problem of antibiotic resistance, the eradication rate of *H. pylori* has gradually declined. On the other hand, quadruple therapy mainly targets the infectious factor and has limited effects on improving structural damage such as gastric mucosal atrophy and intestinal metaplasia. Moreover, it is often accompanied by adverse reactions such as gastrointestinal discomfort and intestinal microbiota imbalance, which may affect patient compliance and overall therapeutic outcomes. Therefore, exploring comprehensive treatment strategies that can both eliminate etiological factors and promote mucosal repair has become a current research focus.

In recent years, the application of oral Chinese patent medicines (OCPMs) in the comprehensive treatment of CAG has attracted increasing attention. Modern pharmacological studies have shown that these medicines can not only inhibit the growth of *H. pylori* but also regulate gastrointestinal hormone levels [such as gastrin (GAS) and motilin (MTL)], improve the secretion of pepsinogens [pepsinogen I (PGI), pepsinogen II (PGII), and their ratio], inhibit the release of inflammatory factors [such as C-reactive protein (CRP), interleukin-6 (IL-6), and tumor necrosis factor-*α* (TNF-α)], and promote gastric mucosal epithelial repair and angiogenesis. Through these mechanisms, they exert synergistic effects in symptom relief, mucosal repair, and delaying disease progression (6).

Further studies have shown that the occurrence and development of CAG are not only related to *H. pylori* infection but also involve multiple mechanisms, including persistent activation of inflammatory responses, enhanced oxidative stress, imbalance of the immune microenvironment, and dysregulation of apoptosis and regeneration (7). Sustained elevation of inflammatory factors can aggravate gastric mucosal injury, while changes in gastrointestinal hormones and pepsinogen levels can reflect the functional status and degree of atrophy of the gastric mucosa (8). Therefore, a comprehensive evaluation of therapeutic effects from multiple dimensions—including *H. pylori* eradication, gastrointestinal hormones, pepsinogens, and inflammatory factors—is of great significance for fully assessing the clinical value of interventions.

At present, several randomized controlled trials (RCTs) have reported certain advantages of oral Chinese patent medicines combined with quadruple therapy in the treatment of CAG. However, existing studies still have limitations, such as small sample sizes, variable study quality, heterogeneity in interventions and treatment durations, and lack of standardized outcome measures. Moreover, high-quality studies comparing different combination regimens and ranking their efficacy are still lacking.

Based on the above background, this study systematically searched relevant domestic and international literature and included RCTs. Traditional meta-analysis and network meta-analysis (NMA) were conducted to evaluate the efficacy and safety of different oral Chinese patent medicines combined with quadruple therapy in the treatment of chronic atrophic gastritis. A comprehensive analysis was performed across multiple dimensions, including *H. pylori* eradication rate, gastrointestinal hormone levels, pepsinogen indices, changes in inflammatory factors, and adverse reactions. Meanwhile, by constructing an evidence network and applying the SUCRA ranking method, the relative advantages of different treatment regimens were compared, with the aim of providing more reliable evidence-based support for optimizing clinical treatment strategies and rational drug use.

## 2 Materials and methods

### 2.1 Study design and registration

This study is a systematic review and Meta-analysis, combined with a network Meta-analysis approach to perform indirect comparisons among different intervention strategies. The study design and reporting followed the Preferred Reporting Items for Systematic Reviews and Meta-Analyses (PRISMA) statement. The study protocol was predesigned in accordance with systematic review standards and registered on the PROSPERO platform with the registration number CRD420261366962.

### 2.2 Literature search strategy

**Figure 1:**
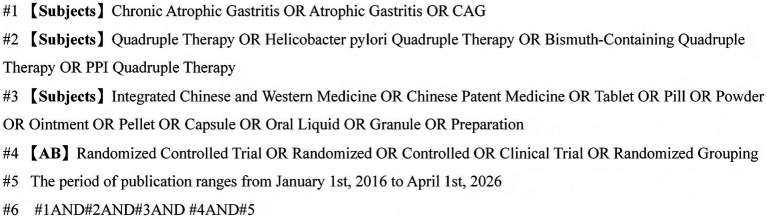
CNKI literature search strategy.

Relevant studies published from January 1, 2016 to April 1, 2026 were systematically searched in the following databases: Chinese databases including China National Knowledge Infrastructure (CNKI), Wanfang Data, VIP Database, and China Biomedical Literature Database (CBM); and English databases including PubMed, Cochrane Library, and Web of Science Core Collection. A combination of subject terms and free-text terms was used for the search. The search strategy mainly included: (1) disease-related terms: “chronic atrophic gastritis,” “atrophic gastritis,” “CAG”; (2) intervention-related terms: “integrated traditional Chinese and Western medicine,” “Chinese patent medicine,” “tablet,” “pill,” “powder,” “paste,” “dan,” “capsule,” “oral liquid,” “granule”; (3) treatment-related terms: “quadruple therapy,” “*Helicobacter pylori* quadruple therapy,” “bismuth-containing quadruple therapy,” “PPI-based quadruple therapy”; and (4) study type-related terms: “randomized controlled trial,” “randomized,” “controlled,” “random allocation,” “RCT,” and “randomized controlled trial.” The search strategies were appropriately adjusted according to the characteristics of each database, and additional studies were identified by screening the reference lists of relevant articles. Taking CNKI as an example, after literature retrieval, the results were imported into Note Express for literature management (Figure 1).

### 2.3 Inclusion and exclusion criteria

#### 2.3.1 Inclusion criteria

The criteria were defined according to the PICOS principle: (1) Participants (P): Patients diagnosed with chronic atrophic gastritis, with no restrictions on sex, age, or disease duration; (2) Interventions (I): Oral Chinese patent medicine combined with quadruple therapy; (3) Comparators (C): Quadruple therapy alone or comparisons between different Chinese patent medicine combination regimens; (4) Outcomes (O): At least one of the following outcomes: *H. pylori* eradication rate; gastrointestinal hormones (GAS, MTL); pepsinogens (PGI, PGII, PGI/PGII); inflammatory markers (CRP, IL-6, TNF-*α*, IL-8, IL-1β); incidence of adverse reactions; (5) Study design (S): Randomized controlled trials (RCTs), published in Chinese or English.

#### 2.3.2 Exclusion criteria

(1) Non-randomized controlled studies (e.g., retrospective studies, case reports); (2) Animal experiments or basic mechanistic studies; (3) Incomplete data or data that could not be extracted; (4) Duplicate publications; (5) Studies involving non-oral traditional Chinese medicine interventions (e.g., acupuncture, massage); (6) Patients with severe comorbidities (e.g., malignant tumors, severe hepatic or renal dysfunction); (7) Studies with obvious methodological flaws.

### 2.4 Study selection and data extraction

Two researchers independently screened the literature. First, titles and abstracts were reviewed for preliminary screening; second, full texts were assessed to determine final inclusion. Discrepancies were resolved through discussion or adjudicated by a third reviewer when necessary. A predesigned data extraction form was used to collect information, including: basic study information (author, year, study type); patient characteristics (sample size, age, disease duration); intervention details (type of Chinese patent medicine, treatment duration); control measures; outcome data; and adverse events.

### 2.5 Risk of Bias assessment

The Cochrane Risk of Bias tool was used to evaluate the quality of included studies, including the following domains: randomization process, allocation concealment, blinding, and completeness of outcome data, selective reporting, and other sources of bias. Each domain was rated as “low risk,” “high risk,” or “unclear risk.”

### 2.6 Statistical analysis

Data analysis was performed using RevMan 5.4 and Stata 18.0 software. (1) Effect size: Relative risk (RR) with 95% confidence intervals (CI) was used for dichotomous variables; mean difference (MD) or standardized mean difference (SMD) was used for continuous variables. (2) Heterogeneity analysis: Chi-square (χ^2^) test and *I*^2^ statistic were applied. If *I*^2^ < 50%, a fixed-effect model was used; otherwise, if *I*^2^ ≥ 50%, a random-effects model was applied, followed by further analysis. (3) Network Meta-analysis: R 4.5.2 and Stata 18.0 were used to perform the network Meta-analysis. A total of 3 Markov chains were set, with 1,000 adaptive iterations (n.adapt), 1,000 burn-in iterations (n.burnin), and a total of 10,000 iterations (n.iter). After excluding the burn-in period samples, the effective posterior sample size was 9,000, with a thinning interval of 1. Evidence network plots were constructed, and consistency was assessed using both global and local methods. Evidence network plots were constructed, and consistency was assessed using both global and local methods. If consistency was acceptable, a consistency model was applied, and SUCRA values were used to rank different interventions. (4) Publication bias: Funnel plots and Egger’s test were used for assessment. Because most evidence networks were star-shaped and lacked closed loops, indirect comparisons were mainly generated through a common comparator (QT). Therefore, the results of the network meta-analysis, particularly SUCRA-based rankings, should be interpreted as exploratory rather than confirmatory evidence.

## 3 Results

### 3.1 Literature search results

**Figure 2:**
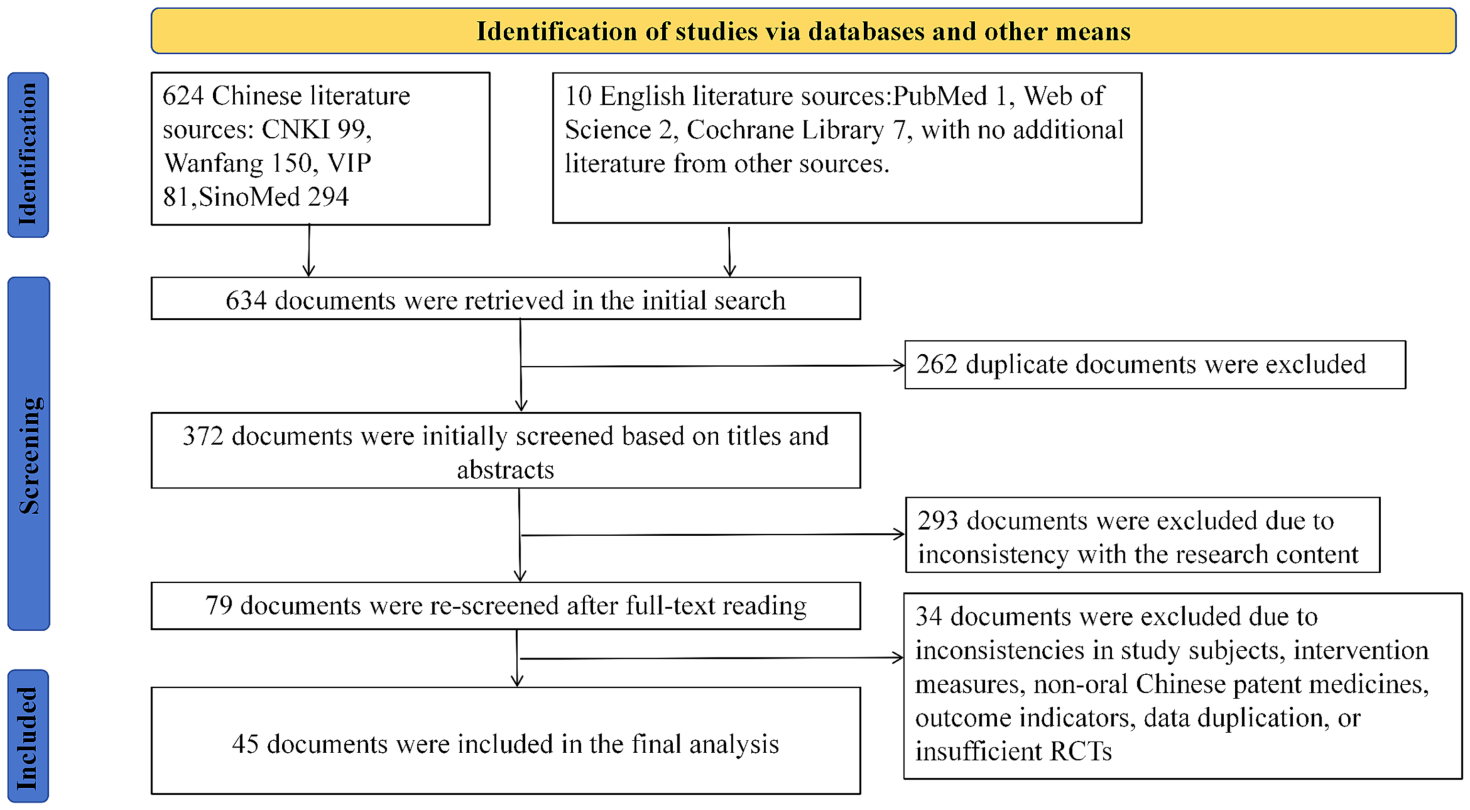
Literature screening process.

A total of 634 studies were retrieved from the above databases according to the search strategy, including 624 Chinese-language articles and 10 English-language articles. Based on the inclusion and exclusion criteria, 589 studies were excluded, and a total of 45 studies were finally included, all of which were Chinese-language publications (Figure 2).

### 3.2 Basic characteristics of included studies

A total of 45 studies (9–53) were included, all of which were two-arm trials, involving 4,465 patients in total, with 2,235 patients in the experimental group and 2,230 patients in the control group. A total of 10 types of oral Chinese patent medicines were involved, including Weifuchun Tablets (WFCT) in 7 studies; Weisu Granules (WSG) and Yangwei Granules (YWG) and Moluo pills (MLP) in 6 studies each; Zhizhu Kuanzhong Capsules (ZZKZC), Jinghua Weikang Capsules (JHWKC), and Biling Weitong Granules (BLWTG) in 4 studies each; and Wumei Pills (WMP), Sanqi Shengwei Pills (SQSWP) in 3 studies each, and Kangfuxin Liquid (KFXL) in 2 studies (Table 1).

**Table 1:** Basic characteristics of included literature.

First author and year	Sample size (*n*)		Age (years)		Disease duration (years)		Treatment group intervention	Control group intervention	Treatment course	Outcome measures
	T	C	T	C	T	C				
Zhao et al., 2018 (9)	79	79	50.83 ± 11.09	51.2 ± 10.99	4.15 ± 1.92	4.31 ± 2.01	YWG + QT	QT	3 months	①②④⑤⑥
Zhang et al., 2019 (10)	41	40	45.83 ± 7.03	46.25 ± 6.58	3.88 ± 1.08	3.94 ± 0.72	YWG + QT	QT	90 days	①②③④⑤⑥⑫
Zhu et al., 2020 (11)	30	30	58.6 ± 10.1	59.6 ± 14.9	1.68 ± 0.19	1.85 ± 0.47	YWG + QT	QT	6 weeks	①⑫
Xiong et al., 2023 (12)	38	38	48.37 ± 6.68	48.29 ± 6.72	5.09 ± 1.56	5.07 ± 1.53	YWG + QT	QT	3 months	①②④⑤⑥
Liu, 2023 (13)	150	150	52.31 ± 11.08	51.06 ± 11.11	4.08 ± 1.84	4.15 ± 1.89	YWG + QT	QT	2 weeks	②
Jia, 2023 (14)	49	49	49.42 ± 7.8	49.51 ± 7.97	—	—	YWG + QT	QT	12 weeks	①②③④
Liang et al., 2019 (15)	50	49	46.6 ± 7.6	47.8 ± 8	4.9 ± 1.1	5.0 ± 1.0	WSG + QT	QT	90 days	①⑧⑫
Qi, 2021 (16)	58	58	45.23 ± 6.12	46.75 ± 7.24	—	—	WSG + QT	QT	2 weeks	⑫
Huang et al., 2021 (17)	50	50	45.11 ± 5.79	45.38 ± 6.64	3.69 ± 0.84	3.72 ± 0.80	WSG + QT	QT	2 weeks	①④⑤⑥⑦⑧⑫
Wang et al., 2022 (18)	60	60	57.65 ± 5.34	57.57 ± 5.28	3.51 ± 0.76	3.46 ± 0.82	WSG + QT	QT	2 weeks	①⑫
Jin, 2023 (19)	39	39	62.07 ± 6.33	61.89 ± 6.85	—	—	WSG + QT	QT	2 weeks	③④⑤⑥
Zhang, 2024 (20)	50	50	45.27 ± 2.20	45.36 ± 2.28	3.36 ± 1.10	3.25 ± 1.08	WSG + QT	QT	1 month	①⑦⑧⑫
Chen, 2025 (21)	31	31	34.56 ± 1.52	34.57 ± 1.54	2.24 ± 0.52	2.26 ± 0.53	WSG + QT	QT	15 days	③⑦⑨⑫
Ma, 2020 (22)	30	30	47.78 ± 3.08	45.43 ± 3.59	4.42 ± 1.29	4.12 ± 1.09	WFCT+QT	QT	1 month	①
Wang, 2020 (23)	35	35	49.79 ± 2.63	49.82 ± 2.54	5.52 ± 1.34	5.36 ± 1.25	WFCT+QT	QT	8 weeks	⑧⑨⑩⑫
Munila et al., 2021 (24)	30	30	49.81 ± 7.22	49.0 ± 6.83	2.34 ± 0.23	2.35 ± 0.24	WFCT+QT	QT	1 month	⑫
Wang, 2021 (25)	49	49	47.23 ± 7.85	48.10 ± 7.92	—	—	WFCT+QT	QT	3 months	①②③⑦⑧
Hu, 2021 (26)	42	42	56.49 ± 5.71	56.54 ± 5.67	7.18 ± 1.54	7.24 ± 1.63	WFCT+QT	QT	3 months	③⑫
Wang et al., 2024 (27)	40	40	39.78 ± 5.34	40.15 ± 6.17	5.75 ± 1.48	5.66 ± 1.35	WFCT+QT	QT	2 weeks	①⑨⑫
Xiao, 2019 (28)	46	46	54.1 ± 2.7	52.3 ± 2.5	1.4 ± 1.0	1.6 ± 1.2	MLP + QT	QT	14 days	①⑫
Wang, 2020 (29)	92	92	53.08 ± 3.61	52.39 ± 0.75	2.50 ± 0.82	2.30 ± 0.84	MLP + QT	QT	14 days	①⑫
Chen, 2024 (30)	48	48	52.84 ± 8.92	52.61 ± 9.35	-	-	MLP + QT	QT	30 days	④⑤⑧⑨⑪
Li, 2024 (31)	80	80	49.26 ± 5.81	48.76 ± 5.96	6.13 ± 1.39	5.92 ± 1.34	MLP + QT	QT	2 weeks	①③④⑤⑧⑨⑩⑪⑫
Shang et al., 2025 (32)	43	43	51.47 ± 9.75	49.88 ± 9.62	3.44 ± 0.91	3.62 ± 0.96	MLP + QT	QT	2 weeks	①③④⑤⑧⑨⑪
Wu et al., 2025 (33)	48	48	50.32 ± 7.05	49.72 ± 6.31	3.68 ± 1.03	3.14 ± 0.77	MLP + QT	QT	14 days	④⑤⑦⑧⑫
Li et al., 2018 (34)	40	40	46.1 ± 5.9	45.5 ± 6.2	7.2 ± 5.1	6.3 ± 4.2	ZZKZC+QT	QT	1 month	①⑦⑨⑩
Li et al., 2019 (35)	40	40	—	—	—	—	ZZKZC+QT	QT	14 days	①⑫
Li et al., 2020 (36)	40	40	45.57 ± 3.45	45.56 ± 3.47	6.20 ± 4.16	6.22 ± 4.15	ZZKZC+QT	QT	1 month	⑦⑨⑩
Liu et al., 2022 (37)	36	36	68.56 ± 3.42	68.34 ± 3.52	2.77 ± 0.56	2.81 ± 0.54	ZZKZC+QT	QT	8 weeks	①⑦⑨⑩⑫
Guan et al., 2022 (38)	41	41	49.86 ± 6.31	50.36 ± 6.47	3.97 ± 1.02	4.05 ± 1.04	JHWKC+QT	QT	4 weeks	⑧⑫
Ni et al., 2023 (39)	60	60	35.65 ± 3.63	35.63 ± 2.01	—	—	JHWKC+QT	QT	2 weeks	①④⑤⑧⑫
Li et al., 2023 (40)	60	60	54.95 ± 12.04	55.69 ± 11.38	5.76 ± 0.73	5.91 ± 0.78	JHWKC+QT	QT	15 days	①④⑤⑫
Li et al., 2025 (41)	77	73	35.89 ± 4.13	36.73 ± 3.41	—	—	JHWKC+QT	QT	2 weeks	①⑫
Lu et al., 2020 (42)	41	41	50.21 ± 10.05	48.93 ± 9.78	7.42 ± 4.93	6.97 ± 4.21	BLWTG+QT	QT	6 months	①⑫
Hong, 2021 (43)	55	55	42.16 ± 3.22	42.59 ± 3.38	3.86 ± 0.91	3.82 ± 0.96	BLWTG+QT	QT	4 months	①⑨⑫
Su et al., 2023 (44)	36	36	48.4 ± 4.5	45.3 ± 5.2	5.05 ± 1.32	4.85 ± 1.20	BLWTG+QT	QT	2 weeks	①⑧⑫
Gong et al., 2024 (45)	56	56	45.21 ± 6.13	44.82 ± 5.7	5.03 ± 1.15	4.83 ± 1.02	BLWTG+QT	QT	8 weeks	①④⑤⑥⑧⑪⑫
Sun et al., 2023 (46)	40	40	51.21 ± 4.83	50.65 ± 4.71	3.86 ± 0.81	3.74 ± 0.75	WMP + QT	QT	2 weeks	①⑧⑪⑫
Mao et al., 2024 (47)	30	30	42.7 ± 1.3	42.17 ± 1.33	4.53 ± 0.97	4.07 ± 0.93	WMP + QT	QT	2 weeks	①⑧⑪⑫
Zhao et al., 2025 (48)	39	39	41.30 ± 3.52	41.26 ± 3.61	4.16 ± 0.75	4.11 ± 0.78	WMP + QT	QT	2 weeks	⑧⑨⑫
Huang, 2023 (49)	40	40	55.10 ± 9.789	54.61 ± 9.809	6.5 ± 2.01	6.4 ± 1.98	SQSWP+QT	QT	12 weeks	①④⑤⑫
Zeng, 2024 (50)	35	35	43.12 ± 2.24	43.55 ± 2.21	—	—	SQSWP+QT	QT	2 weeks	⑧⑫
Huang et al., 2025 (51)	48	49	52.55 ± 10.56	53.95 ± 9.03	6.54 ± 2.01	6.43 ± 1.98	SQSWP+QT	QT	12 weeks	①
Sheng, 2019 (52)	38	38	—	—	—	—	KFXL+QT	QT	28 days	①
Zhao, 2020 (53)	75	75	57.2 ± 12.1	56.6 ± 11.8	5.8 ± 2.6	5.5 ± 2.4	KFXL+QT	QT	2 weeks	①④⑤⑥⑦⑧

①H. pylori eradication rate; ②GAS; ③MTL; ④PGI; ⑤PGII; ⑥PGI/PGII; ⑦CRP; ⑧IL-6; ⑨TNF-α; ⑩IL-8; ⑪IL-1β; ⑫Adverse reactions; “—” indicates not mentioned.

### 3.3 Risk of bias assessment of included studies

**Figure 3:**
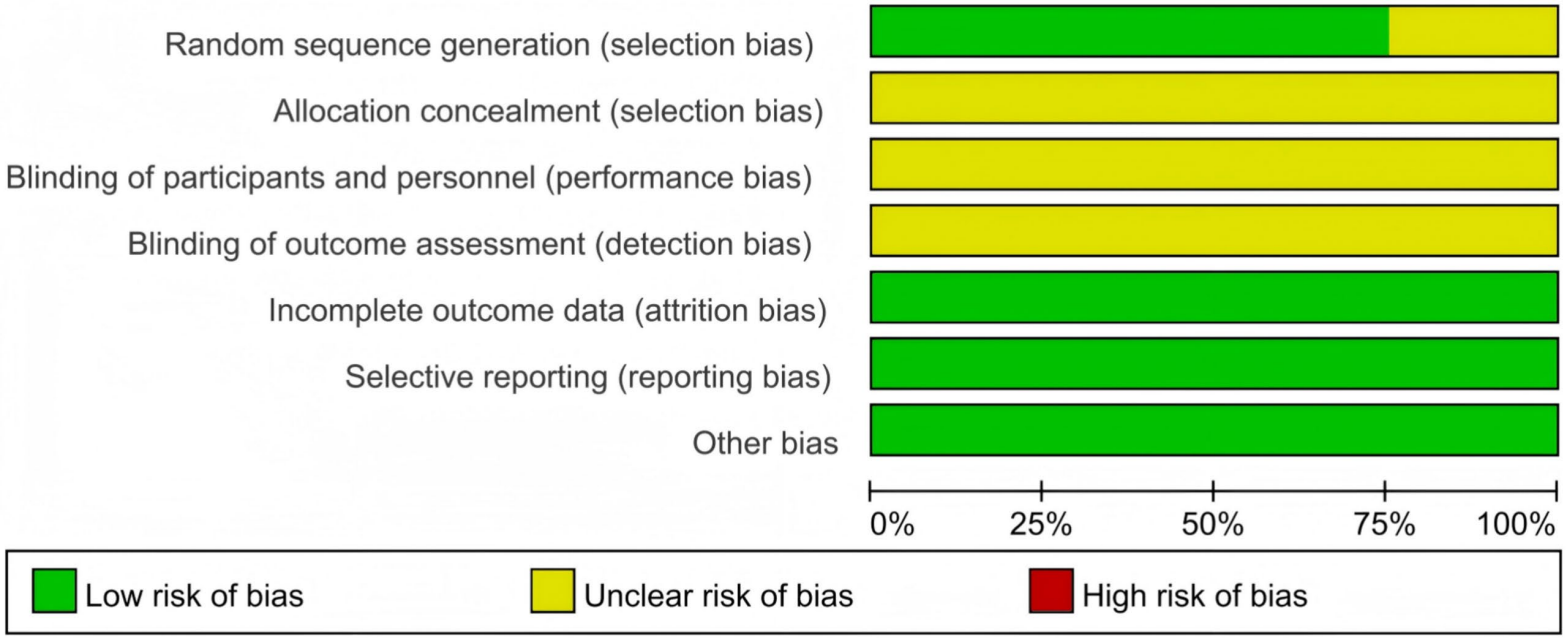
Risk of bias of the included studies.

The risk of bias of the included studies was assessed using the Cochrane Collaboration’s tool implemented in Review Manager 5.4. Among the included studies, 34 trials reported randomization methods. Of these, trials that explicitly described the use of random number tables were judged as low risk for the randomization process. However, trials that only stated the use of an “envelope method” without further specification (e.g., opaque, sequentially numbered, tamper-proof envelopes) were judged as unclear risk, as the lack of detailed description leaves the possibility of selection bias. The remaining studies did not provide detailed descriptions of the randomization process and were also classified as “unclear risk.” None of the included studies provided sufficient information on allocation concealment or blinding procedures; therefore, all were rated as unclear risk in these domains. All studies had complete outcome data without reported dropouts and were considered low risk for incomplete outcome data. All included studies reported the predefined outcome measures, indicating a low risk of selective reporting bias. In addition, no other sources of bias were identified, and all studies were rated as low risk in this domain (Figure 3, Supplementary Figure S1).

### 3.4 Evidence network

**Figure 4:**
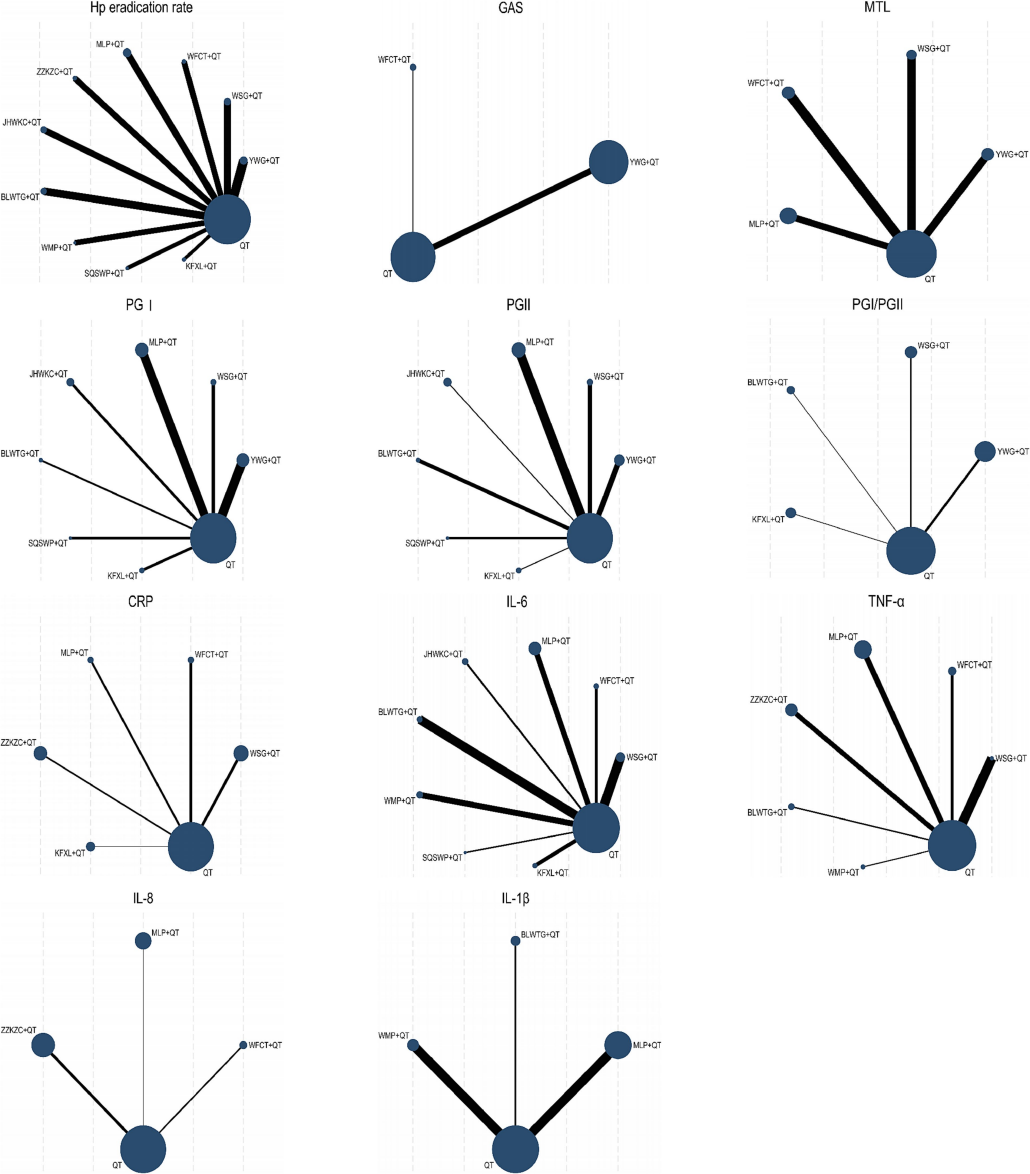
Network evidence plots for all outcome indicators.

Network evidence plots (Figure 4) were generated for all outcome measures of the 10 oral Chinese patent medicines used in the treatment of CAG. In the plots, nodes represent the different interventions; the size of each node is proportional to the sample size, with larger nodes indicating a greater number of patients included in the corresponding treatment. Lines represent direct comparisons between pairs of interventions, and the thickness of each line corresponds to the number of included comparative studies, with thicker lines indicating more studies comparing the two interventions.

### 3.5 Results of network meta-analysis

#### 3.5.1 Inconsistency test

As shown in Figure 4, no closed loops were formed in the evidence networks for the included outcomes. Therefore, formal inconsistency testing could not be performed. In this situation, indirect comparisons were mainly based on star-shaped evidence structures connected through the common comparator (QT). Although network meta-analysis can still be conducted under such conditions, the lack of closed loops limits the ability to evaluate consistency between direct and indirect evidence, thereby reducing the robustness and credibility of comparative estimates and SUCRA rankings. Accordingly, the findings should be interpreted cautiously.

#### 3.5.2 Model convergence assessment

Based on the Markov chain Monte Carlo algorithm, the iterative convergence assessment demonstrated that all Potential Scale Reduction Factor (PSRF) values were less than 1.1, which not only indicates satisfactory model convergence but also confirms that the computational prerequisites for the network meta-analysis have been met.

#### 3.5.3 *H. pylori* eradication rate

**Figure 5:**
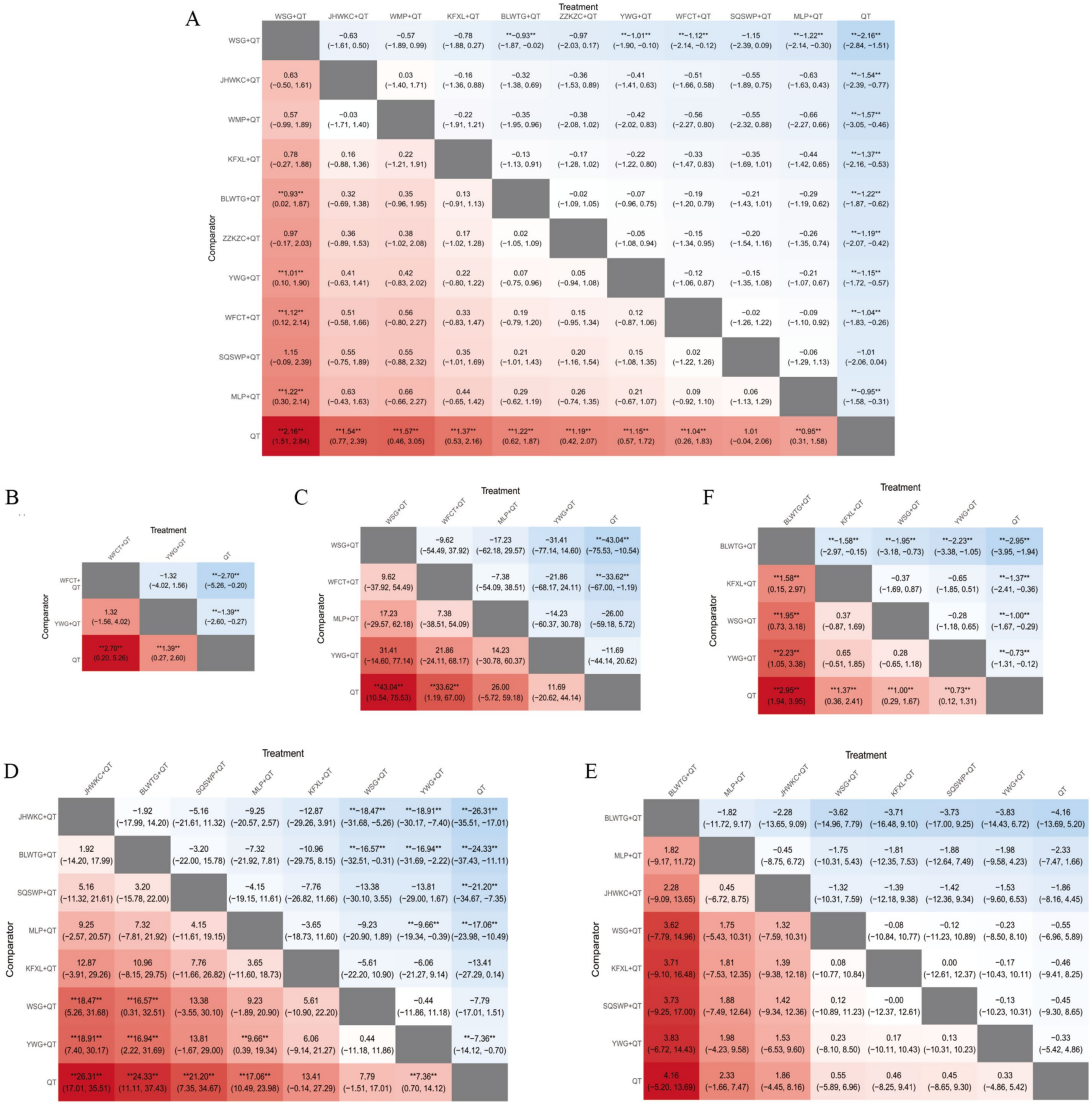
Heatmaps of *H. pylori* eradication rate, Gastrin and Pepsinogen outcomes. **(A)** *H. pylori* eradication rate; **(B)** CAG; **(C)** MTL; **(D)** PGI; **(E)** PGII; **(F)** PGI/PGII. **Indicates a statistically significant difference between the two interventions (*p* < 0.01).

A total of 32 studies (9–12, 14, 15, 17, 18, 20, 22, 25, 27–29, 31, 32, 34, 35, 37, 39–47, 49, 51–53) were included, involving 10 OCPMs+QT and 3,193 patients. There was low statistical heterogeneity across studies (*I*^2^ = 24.9%, *p* = 0.1031) (Supplementary Figure S2). The NMA results (Figure 5A) showed that most combination therapies were more effective than QT alone. Among them, WSG + QT demonstrated superior efficacy compared with some other interventions, with statistically significant differences. However, in the overall pairwise comparisons among the combination regimens, most differences were not statistically significant, suggesting limited differences in efficacy among the interventions.

#### 3.5.4 Gastrin outcome measures

##### 3.5.4.1 Gas

A total of 6 studies (9, 10, 12–14, 25) were included, involving 2 OCPMs+QT and 811 patients. There was high statistical heterogeneity across studies (*I*^2^ = 88.1%, *p* < 0.001) (Supplementary Figure S3). The NMA results (Figure 5B) showed that combination therapies were overall more effective than QT alone.

##### 3.5.4.2 MTL

A total of 8 studies (10, 14, 19, 21, 25, 26, 31, 32) were included, involving 4 OCPMs combined with QT and 747 patients. There was high statistical heterogeneity across studies (*I*^2^ = 91.8%, *p* < 0.001) (Supplementary Figure S4). The NMA results (Figure 5C) showed that, in comparisons with QT, WSG + QT and WFCT+QT demonstrated a statistically significant difference, while the other combination therapies showed a trend toward improvement but did not reach statistical significance. Meanwhile, no statistically significant differences were observed in the pairwise comparisons among the combination regimens.

#### 3.5.5 Pepsinogen outcome measures

##### 3.5.5.1 PGI

A total of 16 studies (9, 10, 12, 14, 17, 19, 30–33, 39, 40, 45, 49, 53) were included, involving 7 OCPMs combined with QT and 1,611 patients. There was high statistical heterogeneity across studies (*I*^2^ = 97.8% *p* < 0.001) (Supplementary Figure S5). The NMA results (Figure 5D) showed that, compared with QT alone, most combination therapies (such as JHWKC+QT, BLWTG+QT, SQSWP+QT, and MLP + QT) demonstrated superior efficacy, with some comparisons reaching statistical significance.

Furthermore, in the pairwise comparisons among the combination regimens, certain interventions (such as JHWKC+QT and BLWTG+QT) also showed advantages over other treatments, with statistically significant differences. Overall, these findings suggest that OCPMs combined with QT are generally more effective than QT alone, and there are some differences in efficacy among the combination regimens.

##### 3.5.5.2 PGII

A total of 14 studies (9, 10, 12, 17, 19, 30–33, 39, 40, 45, 49, 53) were included, involving 7 OCPMs combined with QT and 1,513 patients. There was high statistical heterogeneity across studies (*I*^2^ = 87.5%, *p* < 0.001) (Supplementary Figure S6). The NMA results (Figure 5E) showed that, although all OCPMs combined with QT tended to improve outcomes compared with QT alone, none of the differences reached statistical significance. Similarly, no significant differences were observed among the combination regimens.

##### 3.5.5.3 PGI/PGII

A total of 7studies (9, 10, 12, 17, 19, 45, 53) were included, involving 4 OCPMs combined with QT and 755 patients. There was high statistical heterogeneity across studies (*I*^2^ = 92.5%, *p* < 0.001) (Supplementary Figure S7). The NMA results (Figure 5F) showed that, compared with QT alone, BLWTG+QT, WSG + QT, and YWG + QT demonstrated superior efficacy, with statistically significant differences. In the pairwise comparisons among the combination regimens, some interventions (such as BLWTG+QT) also showed advantages over others. Overall, these findings suggest that OCPMs combined with QT are generally more effective than QT alone, with some differences among the regimens.

#### 3.5.6 Inflammatory factor indicators

##### 3.5.6.1 CRP

**Figure 6:**
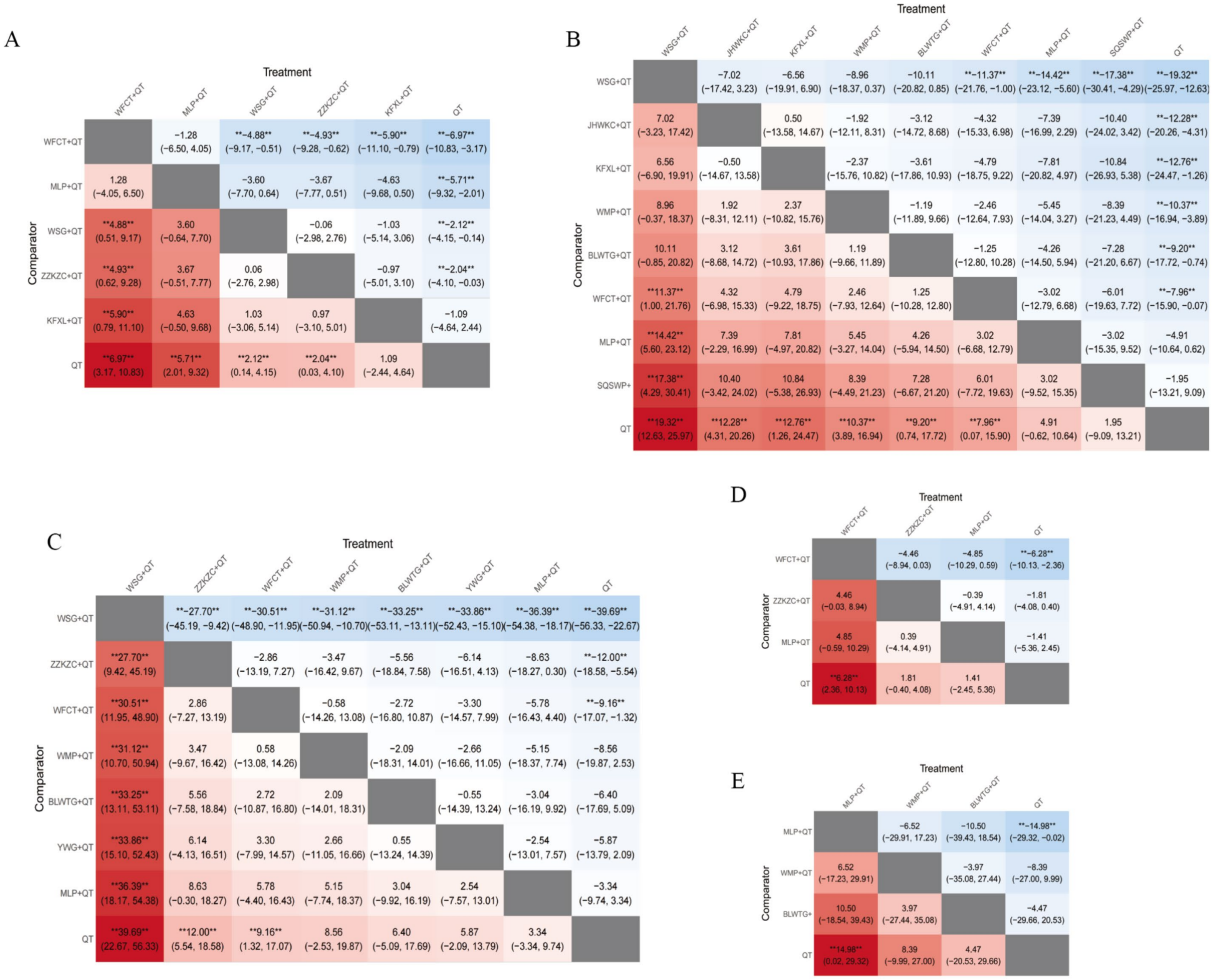
Heatmaps of inflammatory factor indicators. **(A)** CRP; **(B)** IL-6; **(C)** TNF-*α*; **(D)** IL-8; **(E)** IL-1β. **Indicates a statistically significant difference between the two interventions (*p* < 0.01).

A total of 9 studies (20, 23, 24, 29, 37, 38, 40, 41, 53) were included, involving 5 OCPMs combined with quadruple therapy (OCPMs+QT) and 838 patients. There was high statistical heterogeneity across studies (*I*^2^ = 96.1%, *p* < 0.001) (Supplementary Figure S8). The NMA results (Figure 6A) showed that, compared with QT alone, WFCT+QT, MLP + QT, WSG + QT, and ZZKZC+QT significantly reduced CRP levels, while KFXL+QT showed a decreasing trend but did not reach statistical significance. In the pairwise comparisons among the combination regimens, WFCT+QT demonstrated superior efficacy in some comparisons, with a greater reduction in CRP levels than WSG + QT, ZZKZC+QT, and KFXL+QT, and the differences were statistically significant. Most of the other comparisons among the combination therapies did not show statistically significant differences. Overall, these findings suggest that OCPMs combined with quadruple therapy are more effective than QT alone in reducing the inflammatory marker CRP, with WFCT+QT showing relatively greater anti-inflammatory efficacy.

##### 3.5.6.2 Il-6

A total of 18 studies (15, 17, 20, 23, 25, 30–33, 38, 39, 44–48, 50, 53) were included, involving 8 OCPMs combined with quadruple therapy (OCPMs + QT) and 1,792 patients. There was high statistical heterogeneity across studies (*I*^2^ = 99.1%, *p* < 0.001) (Supplementary Figure S9). The NMA results (Figure 6B) showed that, compared with quadruple therapy (QT) alone, most OCPMs combined with QT (such as WSG + QT, JHWKC+QT, KFXL+QT, WMP + QT, BLWTG+QT, and WFCT+QT) demonstrated superior efficacy, with statistically significant differences. In the pairwise comparisons among the combination regimens, WSG + QT was superior to some other interventions, suggesting differences in efficacy among the regimens. Overall, all combination therapies were more effective than QT alone.

##### 3.5.6.3 TNF-*α*

A total of 11 studies (21, 23, 27, 30–32, 34, 36, 37, 43, 48) were included, involving 6 OCPMs combined with quadruple therapy (OCPMs + QT) and 974 patients. There was high statistical heterogeneity across studies (*I*^2^ = 92.8%, *p* < 0.001) (Supplementary Figure S10). The NMA results (Figure 6C) showed that, compared with QT alone, only WSG + QT and ZZKZC+QT demonstrated statistically significant reductions in TNF-α levels. Furthermore, WSG + QT was superior to some other OCPMs combined regimens in pairwise comparisons, with statistically significant differences. Overall, these findings suggest that there are differences in anti-inflammatory effects among the combination therapies, with WSG + QT showing relatively greater efficacy.

##### 3.5.6.4 Il-8

A total of 5 studies (23, 31, 34, 36, 37) were included, involving 3 OCPMs combined with quadruple therapy (OCPMs+QT) and 462 patients. There was high statistical heterogeneity across studies (*I*^2^ = 95.5%, *p* < 0.001) (Supplementary Figure S11). The NMA results (Figure 6D) showed that, compared with QT alone, only WFCT+QT demonstrated a statistically significant improvement, while ZZKZC+QT and MLP + QT did not show significant differences. In the pairwise comparisons among the combination regimens, no statistically significant differences were observed, suggesting limited differences in efficacy among the interventions.

##### 3.5.6.5 Il-1β

A total of 6 studies (30–32, 45–47) were included, involving 3 OCPMs combined with quadruple therapy (OCPMs + QT) and 594 patients. There was high statistical heterogeneity across studies (*I*^2^ = 96.1%, *p* < 0.001) (Supplementary Figure S12). The NMA results (Figure 6E) showed that, compared with QT alone, only MLP + QT demonstrated a statistically significant improvement, while WMP + QT and BLWTG+QT did not show significant differences. In the pairwise comparisons among the combination regimens, no statistically significant differences were observed, suggesting limited differences in efficacy among the interventions.

#### 3.5.7 Adverse reactions

Analysis of adverse reactions included in 31 studies showed that the observed adverse reactions mainly involved the digestive system, circulatory system, nervous system and skin. Digestive system reactions were the most common, including nausea, vomiting, diarrhea, abdominal distension, constipation, bitter taste, epigastric discomfort and other gastrointestinal symptoms. Palpitations and chest tightness were the main manifestations of the circulatory system; headache, dizziness and somnolence were observed in the nervous system; and skin reactions were dominated by rash and pruritus. Overall, adverse reactions were mild and transient, with no concentrated reports of serious adverse events, indicating a generally favorable safety profile Table 2.

**Table 2:** Incidence of adverse reactions.

First author and year	Adverse reactions	
	Experimental group	Control group
Zhang et al., 2019 (10)	1 case headache, 3 cases mild gastrointestinal reaction, 1 case rash	1 case nausea, 2 cases mild gastrointestinal reaction, 1 case abdominal bloating
Zhu et al., 2020 (11)	1 case bitter oral taste, 1 case mild abdominal pain, 2 cases mild diarrhea	1 case mild diarrhea, 2 cases nausea with retching
Liang et al., 2019 (15)	2 cases mild gastrointestinal reaction, 1 case rash, 2 cases headache	1 case mild gastrointestinal reaction, 1 case nausea, 1 case abdominal bloating
Qi, 2021 (16)	2 cases epigastric discomfort, 1 case nausea and vomiting, 1 case constipation	1 case hepatic function impairment, 6 cases epigastric discomfort, 5 cases nausea and vomiting, 3 cases constipation
Huang et al., 2021 (17)	2 cases abdominal bloating, 1 case nausea, 2 cases gastrointestinal reaction	1 case abdominal bloating, 1 case nausea, 2 cases gastrointestinal reaction
Wang et al., 2022 (18)	3 cases rash, 4 cases diarrhea, 2 cases dry mouth, 1 case abdominal pain, 2 cases fatigue	2 cases rash, 3 cases diarrhea, 3 cases dry mouth, 1 case abdominal pain, 2 cases fatigue
Zhang, 2024 (20)	1 case fever, 1 case rash, 1 case diarrhea	2 cases fever, 1 case rash, 2 cases diarrhea
Chen, 2025 (21)	1 case constipation	1 case constipation, 2 cases nausea and vomiting, 2 cases epigastric discomfort, 1 case hepatic function impairment
Wang, 2020 (23)	1 case allergy, 2 cases transient hepatic function impairment, 2 cases drowsiness	1 case allergy, 1 case transient hepatic function impairment, 1 case drowsiness
Munila et al., 2021 (24)	None	None
Hu, 2021 (26)	1 case allergy, 2 cases transient hepatic function impairment, 2 cases drowsiness	1 case allergy, 2 cases transient hepatic function impairment, 1 case drowsiness
Wang et al., 2024 (27)	None	None
Xiao, 2019 (28)	1 case aggravated nausea	1 case aggravated nausea, 2 cases aggravated abdominal pain, 3 cases headache
Wang, 2020 (29)	3 cases nausea and vomiting, 2 cases headache, 2 cases abdominal pain	2 cases nausea and vomiting, 2 cases headache, 1 case abdominal pain
Li, 2024 (31)	6 cases nausea, 3 cases diarrhea	5 cases nausea, 2 cases diarrhea
Wu et al., 2025 (33)	3 cases nausea and vomiting, 2 cases constipation, 3 cases rash	1 case nausea and vomiting, 2 cases constipation, 2 cases rash
Li et al., 2019 (34)	1 case dry mouth, 1 case bitter oral taste, 1 case nausea	3 cases dry mouth, 2 cases bitter oral taste, 1 case nausea, 1 case dry stool, 1 case constipation, 2 cases black stool
Liu et al., 2022 (37)	1 case nausea, 1 case dry mouth and bitter taste, 1 case constipation	4 cases nausea, 3 cases dry mouth and bitter taste, 3 cases constipation
Guan et al., 2022 (38)	4 cases gastrointestinal symptoms, 2 cases skin symptoms, 3 cases neurological symptoms	3 cases gastrointestinal symptoms, 2 cases skin symptoms, 2 cases neurological symptoms
Ni et al., 2023 (39)	1 case gastrointestinal symptom	1 case gastrointestinal symptom, 3 cases skin symptoms, 3 cases neurological symptoms
Li et al., 2023 (40)	2 cases nausea, 1 case vomiting, 1 case dizziness	1 case nausea, 1 case vomiting, 1 case diarrhea
Li et al., 2025 (41)	1 case gastrointestinal symptom, 2 cases skin symptoms, 1 case neurological symptom	2 cases gastrointestinal symptoms, 3 cases skin symptoms, 5 cases neurological symptoms
Lu et al., 2020 (42)	None	None
Hong, 2021 (43)	2 cases constipation, 3 cases nausea, 4 cases flushing, 5 cases diarrhea	3 cases constipation, 2 cases nausea, 2 cases flushing, 4 cases diarrhea
Su et al., 2023 (44)	1 case abdominal bloating, 1 case nausea	1 case nausea
Gong et al., 2024 (45)	2 cases mild nausea and vomiting	1 case nausea and vomiting
Sun et al., 2023 (46)	1 case dizziness, 2 cases nausea	1 case dizziness, 2 cases nausea, 1 case constipation
Mao et al., 2024 (47)	1 case constipation	3 cases nausea and vomiting, 3 cases constipation, 2 cases pruritus, 2 cases rash
Zhao et al., 2025 (48)	1 case constipation, 1 case nausea and vomiting	2 cases constipation, 2 cases nausea and vomiting, 1 case pruritus, 1 case rash
Huang, 2023 (49)	1 case nausea, 1 case diarrhea	1 case bitter oral taste, 2 cases nausea, 2 cases diarrhea
Zeng, 2024 (50)	1 case nausea, 1 case palpitations	1 case nausea, 1 case palpitations, 1 case chest distress

#### 3.5.8 Ranking based on SUCRA values

**Figure 7:**
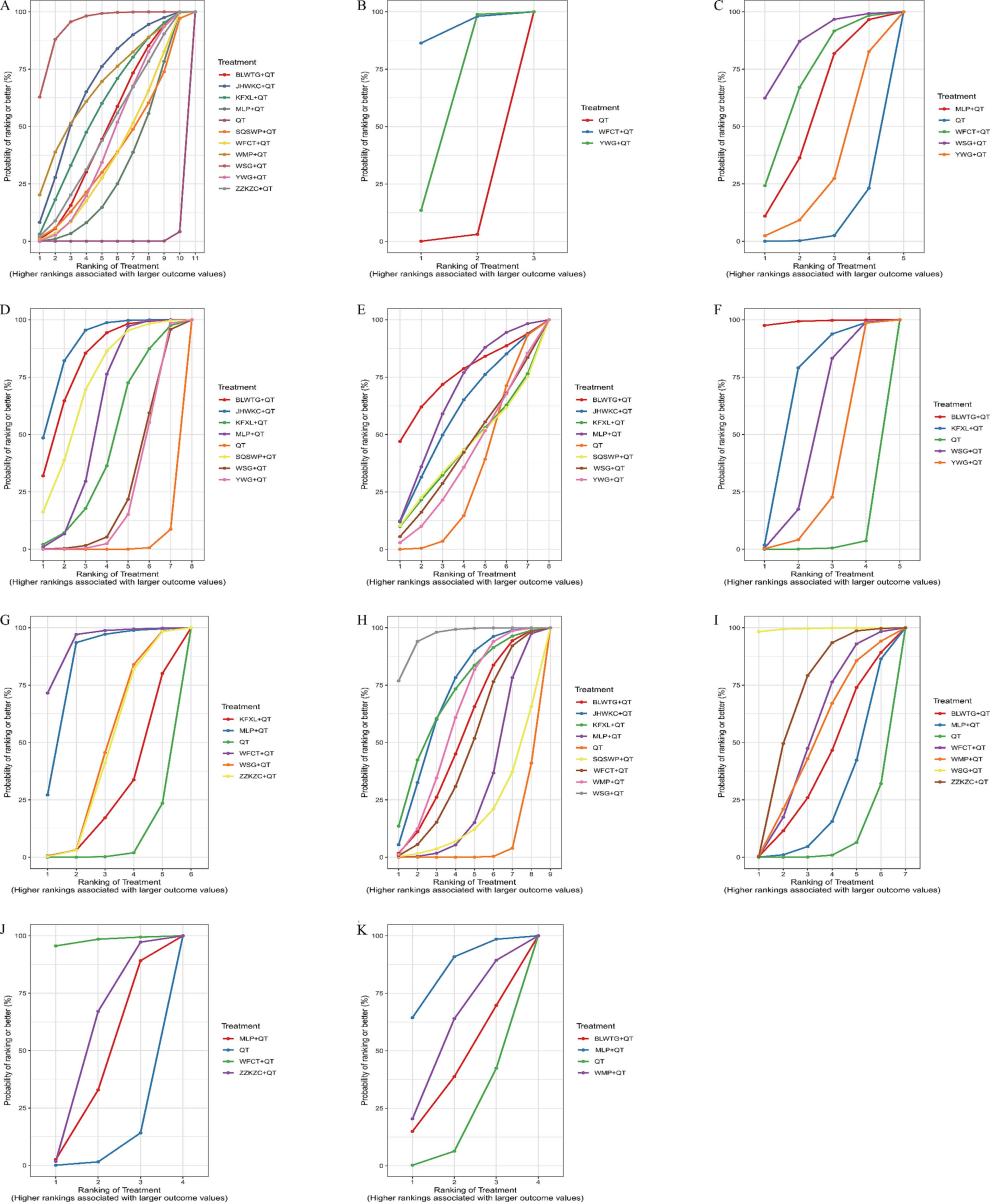
Cumulative probability ranking of OCPMs + QT for the Treatment of CAG. **(A)** *H. pylori* eradication rate; **(B)** GAS; **(C)** MTL; **(D)** PGI; **(E)** PGII; **(F)** PGI/PGII; **(G)** CRP; **(H)** IL-6; **(I)** TNF-α; **(J)** IL-8; **(K)** IL-1β.

It should be emphasized that all SUCRA rankings are exploratory and indirect due to the star-shaped evidence network and absence of head-to-head trials. These results do not represent definitive clinical recommendations. The SUCRA ranking indicated that WSG combined with QT showed the highest SUCRA probability for improving *H. pylori* eradication rate, increasing MTL levels, and reducing IL-6 and TNF-*α* levels. WFCT showed a favorable SUCRA ranking for increasing GAS levels and reducing CRP and IL-8 levels. MLP showed the highest SUCRA ranking for reducing IL-1β levels, while JHWKC showed the highest SUCRA probability for improving PGI levels. In addition, BLWTG ranked highest by SUCRA for improving PGII levels and the PGI/PGII ratio (Figure 7, Table 3). However, these rankings are based on indirect evidence and should be interpreted as exploratory rather than definitive; confirmation in head-to-head trials is required.

**Table 3:** Cumulative probability ranking table of OCPMs combined with QT in the treatment of CAG.

Intervention measures	Hp eradication rate		GAS		MLT		PGI		PGII		PGI/PGII		CRP		IL-6		TNF-α		IL-8		IL-1β	
	SUCRA%	RANK	SUCRA%	RANK	SUCRA%	RANK	SUCRA%	RANK	SUCRA%	RANK	SUCRA%	RANK	SUCRA%	RANK	SUCRA%	RANK	SUCRA%	RANK	SUCRA%	RANK	SUCRA%	RANK
YWG + QT	46	7	54.0	2	29.3	4	23.7	7	37.1	7	27.8	4	—	—	—	—	—	—	—	—	—	—
WSG + QT	95.7	1	—	—	91.2	1	24.8	6	42.4	5	50.3	3	47.1	3	97.4	1	99.9	1	—	—	—	—
WFCT+QT	39.7	9	95.9	1	71.7	2	—	—	—	—	—	—	95.7	1	44.6	5	57.3	3	100	1	—	—
MLP + QT	32.3	10	—	—	55.1	3	58.2	4	67.9	2	—	—	84.3	2	29.4	6	26.6	6	41.7	3	89.2	1
ZZKZC + QT	50.2	6	—	—	—	—	—	—	—	—	—	—	44.9	4	—	—	73.1	2	58.2	2	—	—
JHWKC + QT	69.1	2	—	—	—	—	90.7	1	60.4	3	—	—	—	—	70.8	2	—	—	—	—	—	—
BLWTG + QT	51.8	5	—	—	—	—	83.9	2	77.9	1	100.0	1	—	—	54.4	4	40.9	5	—	—	40.2	3
WMP + QT	64.4	3	—	—	—	—	—	—	—	—	—	—	—	—	60.1	3	52.3	4	—	—	58.3	2
SQSWP + QT	40.3	8	—	—	—	—	73.5	3	40.8	6	—	—	—	—	16.4	7	—	—	—	—	—	—
KFXL + QT	60.2	4	—	—	—	—	44.9	5	42.9	4	71.9	2	25.5	5	72		—	—	—	—	—	—
QT	0.5	11	0.1	3	2.6	5	0.3	8	30.7	8	0	5	2.6	6	4.9	8	3.0	7	0.1	4	12.4	4

#### 3.5.9 Assessment of publication bias

**Figure 8:**
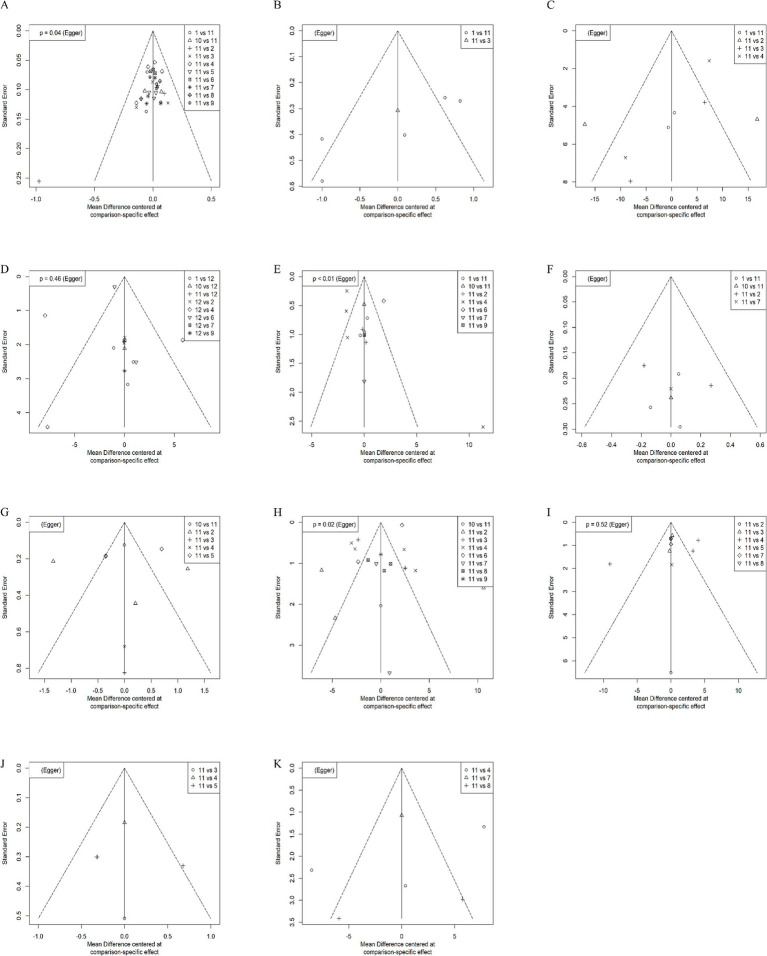
Calibrated funnel plots of various outcome indicators for OCPMs + QT in the Treatment of CAG. **(A)** *H. pylori* eradication rate; **(B)** GAS; **(C)** MTL; **(D)** PGI; **(E)** PGII; **(F)** PGI/PGII; **(G)** CRP; **(H)** IL-6; **(I)** TNF-α; **(J)** IL-8; **(K)** IL-1β.

Publication bias was assessed using comparison-adjusted funnel plots and Egger’s test. For outcomes with a sufficient number of studies (e.g., *H. pylori* eradication rate, PGII, and IL-6), Egger’s test yielded statistically significant results (*p* < 0.05), suggesting potential publication bias. For PGI and TNF-*α*, Egger’s test showed no significant asymmetry (*p* > 0.05). However, for several other outcomes (e.g., GAS, MTL, PGI/PGII, CRP, IL-8, and IL-1β), the number of included studies was limited. Under such conditions, Egger’s test has low statistical power and its results are unreliable for detecting or excluding publication bias. Therefore, while publication bias cannot be ruled out for some outcomes, the overall assessment is limited by the small number of studies available for most outcomes. All findings should be interpreted with caution Figure 8.

#### 3.5.10 Confidence in network meta-analysis (CINeMA) assessment of evidence certainty

The certainty of evidence for the network meta-analysis was assessed using the CINeMA online tool (54–56), which evaluates six domains, including within-study bias, across-study bias, indirectness, imprecision, heterogeneity, and incoherence. The overall certainty of evidence for comparisons among the included interventions was generally low or very low (Supplementary Table S1).

The main reasons for downgrading were as follows. First, the methodological quality of the included studies was suboptimal. Although some trials reported randomization methods, many did not provide sufficient details, and none clearly described allocation concealment or blinding procedures, suggesting a potential risk of selection and performance bias. Second, variations in diagnostic criteria, outcome measures, intervention protocols, and treatment durations across studies may have affected the consistency and comparability of the results, thereby contributing to indirectness and inconsistency. Third, for several outcomes, the number of included studies and sample sizes were limited, resulting in relatively wide confidence intervals and reduced precision. In addition, a certain degree of heterogeneity was observed among studies, indicating variability in treatment effects.

It is also noteworthy that all included randomized controlled trials were conducted in single-center settings and published in Chinese, which may introduce potential regional and publication biases and limit the generalizability of the findings. Furthermore, insufficient reporting of adverse events and long-term outcomes in some studies may affect the completeness of the evidence.

Overall, although the current evidence suggests that oral Chinese patent medicines combined with quadruple therapy may provide clinical benefits in patients with CAG, the strength of evidence remains limited, and the findings should be interpreted with caution. Further high-quality, large-scale, multicenter randomized controlled trials are warranted to confirm these results.

## 4 Summary and discussion

This study employed a NMA to systematically evaluate the efficacy of OCPMs combined with quadruple therapy for CAG. A total of 45 randomized controlled trials were included, involving 10 types of OCPMs. Multiple outcome dimensions were comprehensively assessed, including *H. pylori* eradication rate, gastrointestinal hormone levels, pepsinogen indices, inflammatory markers, and adverse events, with the aim of providing evidence-based guidance for clinical decision-making.

CAG is a chronic inflammatory disease characterized by the loss of gastric glandular structures and is widely recognized as an important precancerous lesion of gastric cancer. Its progression follows a multistep cascade from chronic inflammation to atrophy, intestinal metaplasia, dysplasia, and ultimately carcinoma (57, 58). Within this pathological continuum, multiple processes—including *H. pylori* infection, persistent inflammatory responses, impairment of the gastric mucosal barrier, dysregulation of cell proliferation and apoptosis, and alterations in the local immune microenvironment—interact and reinforce each other, collectively driving disease progression (59, 60). Therefore, the evaluation of therapeutic efficacy in CAG should be based on the integrated improvement of multidimensional outcomes rather than reliance on a single clinical or biochemical endpoint.

From the perspective of TCM, CAG is generally categorized under conditions such as “epigastric pain” and “distention and fullness.” Its pathogenesis is primarily rooted in spleen–stomach deficiency, often accompanied by qi stagnation, blood stasis, and phlegm-dampness. With disease progression, patterns of deficiency of both qi and yin with concomitant excess factors are commonly observed, resulting in a complex condition characterized by both deficiency and excess. Based on the holistic concept and syndrome differentiation, OCPMs exert therapeutic effects through multi-target and multi-pathway mechanisms, including tonifying qi and nourishing yin, promoting blood circulation, regulating qi, harmonizing the stomach, and clearing heat and dampness. These properties confer advantages in alleviating inflammation, promoting mucosal repair, and restoring gastrointestinal function (61, 62).

Currently, a variety of OCPMs have been applied in the clinical management of CAG and have demonstrated favorable effects in improving *H. pylori* eradication rates, enhancing gastric mucosal function, and relieving clinical symptoms. However, the relative efficacy among different OCPMs remains unclear due to the lack of direct comparative evidence. Therefore, a comprehensive evaluation using network meta-analysis is warranted to compare the effectiveness of these interventions and to provide a robust evidence base for optimizing clinical treatment strategies.

### 4.1 Ranking of efficacy and quality of evidence

The NMA showed that, across most outcomes, OCPMs combined with QT generally demonstrated superior efficacy compared with quadruple therapy alone. Based on SUCRA probability rankings, different OCPMs exhibited varying advantages across specific outcome measures.

Specifically, WSG combined with QT showed relatively favorable rankings in improving *H. pylori* eradication rate, increasing MTL levels, and reducing inflammatory markers such as IL-6 and TNF-*α*. WFCT demonstrated advantages in increasing GAS levels and reducing inflammatory indicators, including CRP and IL-8. MLP ranked relatively higher in reducing IL-1β levels, while JHWKC showed potential advantages in improving PGI levels. In addition, BLWTG exhibited relatively favorable rankings in improving PGII levels and the PGI/PGII ratio.

Overall, different OCPMs appear to exert their therapeutic advantages across multiple pathological dimensions, including *H. pylori* infection, inflammatory response, and gastric mucosal function, suggesting that differences in efficacy may be related to their distinct mechanistic focuses. According to the CINeMA assessment, the quality of evidence for these outcomes varied, with most being rated as low or very low. The main limitations were related to risk of bias, imprecision, and heterogeneity among the included studies. Therefore, the SUCRA-based rankings should be interpreted with caution, taking into account the underlying quality of evidence.

### 4.2 Preclinical mechanistic evidence

Clinical and experimental studies have shown that the therapeutic effects of various OCPMs in CAG primarily target key pathological processes, including *H. pylori* infection, chronic inflammation, gastric mucosal injury and repair, and gastrointestinal dysfunction. The mechanisms of these agents are characterized by multi-target and multi-pathway synergistic regulation.

#### 4.2.1 Anti-*H. pylori* effects and inhibition of inflammatory response

*H. pylori* infection is a major driver of CAG development and progression. Several OCPMs exert therapeutic effects by inhibiting *H. pylori* colonization and reducing inflammatory factor levels. Both clinical and experimental studies have demonstrated that JHWKC and BLWTG possess anti-*H. pylori* activity, which may be attributed to inhibition of bacterial adhesion, disruption of biofilm formation, and reduction of bacterial load. Concurrently, these agents downregulate pro-inflammatory cytokines such as TNF-*α*, IL-6, and IL-1β, thereby alleviating gastric mucosal inflammation (63–65). Additionally, some OCPMs may ameliorate *H. pylori*-related chronic inflammation by modulating inflammation-related signaling pathways such as NF-κB and reducing CRP and IL-6 levels (66). Collectively, these mechanisms act on the “*H. pylori*–inflammation–mucosal injury” axis and may help delay CAG progression.

#### 4.2.2 Anti-inflammatory effects and regulation of inflammation–carcinogenesis pathways

Persistent inflammation and the activation of related signaling pathways are critical mechanisms underlying the progression from CAG to intestinal metaplasia and gastric cancer. Studies have shown that WFCT inhibits inflammatory signaling pathways such as NF-κB, reduces TNF-α and IL-8 levels, and improves gastric mucosal histopathology, suggesting potential effects in reversing atrophy and intestinal metaplasia (67, 68). MLP may exert its effects by regulating the PI3K/Akt and TNF signaling pathways, thereby inhibiting inflammatory cascades and modulating cell proliferation and apoptosis, which could potentially intervene in the transition from inflammation to precancerous lesions (69). Thus, these agents may not only alleviate inflammation but also play a role in delaying or preventing disease progression.

#### 4.2.3 Promotion of gastric mucosal repair and improvement of glandular function

Gastric gland atrophy and mucosal barrier impairment are key pathological features of CAG. Some OCPMs promote mucosal repair and regulate secretory function. YWG has been shown to increase levels of prostaglandin E2 (PGE2), somatostatin (SS), and gastrin (GAS), improve pepsinogen indices (PGI, PGI/PGII ratio), and reduce inflammatory factors, thereby facilitating mucosal repair and functional recovery (70, 71). KFXL primarily enhances tissue regeneration, promotes epithelial cell proliferation, and improves local microcirculation, thus supporting mucosal repair (72, 73). In addition, Panax notoginseng-based preparations may improve gastric mucosal blood supply and promote tissue regeneration by activating blood circulation, indirectly enhancing mucosal repair capacity. These mechanisms mainly target the “mucosal injury–repair imbalance” process and represent an important approach to improving atrophic changes.

#### 4.2.4 Regulation of gastrointestinal motility and gastric microenvironment

Gastrointestinal motility disorders and disturbances in the gastric microenvironment are common in patients with CAG and may further exacerbate mucosal injury. Some OCPMs exert auxiliary therapeutic effects by regulating gastrointestinal hormones and motility. ZZKZC regulates gastrointestinal hormones such as GAS and MTL, improves gastric emptying, and relieves symptoms like postprandial fullness, thereby optimizing the gastric microenvironment (74, 75). WSG also modulates gastrointestinal function and improves delayed gastric emptying and dyspeptic symptoms (76, 77). Although direct evidence for reversing pathological changes is relatively limited for these agents, they may create favorable conditions for mucosal repair by improving gastrointestinal motility and the local environment.

#### 4.2.5 Integration of mechanistic pathways

In summary, although different OCPMs vary in their specific targets and dominant mechanisms, their overall therapeutic effects can be integrated into a multidimensional regulatory network centered on the pathological axis of “*H. pylori* infection–chronic inflammation–gastric mucosal injury–repair imbalance–precancerous transformation.” Within this framework, anti-*H. pylori* and antimicrobial effects act on the initiating factors of the disease; anti-inflammatory and immunomodulatory effects interrupt inflammatory cascades and reduce ongoing mucosal damage; and mucosal repair together with microcirculation improvement helps restore glandular structure and barrier function. Regulation of gastrointestinal motility and the gastric microenvironment further optimizes conditions for mucosal healing. These mechanisms are not independent but rather interact synergistically to influence key stages of CAG progression.

When considered alongside the results of this network meta-analysis, the differences in efficacy among OCPMs across various outcomes may reflect their distinct roles in different segments of this pathological axis. For example, WFCT and MLP show advantages in regulating inflammation and potentially intervening in inflammation–carcinogenesis processes; YWG and KFXL are more focused on mucosal repair and functional recovery; whereas JHWKC and BLWTG primarily act by inhibiting *H. pylori* and alleviating infection-related inflammation. This correspondence between mechanistic focus and clinical outcomes may, to some extent, explain the relative efficacy rankings observed in this network meta-analysis.

#### 4.2.6 A note on mechanistic interpretation

The mechanistic pathways discussed above (e.g., NF-κB inhibition, PI3K/Akt modulation, anti-biofilm activity, angiogenesis, and immune regulation) are primarily derived from preclinical studies, including *in vitro* experiments and animal models. While these findings provide valuable insights into the potential mechanisms of action of OCPMs, they were not directly measured in the clinical trials included in this meta-analysis. Therefore, the proposed mechanisms should be considered hypotheses to be tested in future research rather than established explanations for the clinical observations in this study.

### 4.3 Clinical implications

This study systematically compared the relative efficacy of different OCPMs combined with quadruple therapy in patients with CAG based on network meta-analysis. It should be noted that, according to the CINeMA assessment, the quality of evidence for most outcomes was low or very low, mainly due to the risk of bias, imprecision, and heterogeneity of the included studies. Therefore, the findings should be interpreted as preliminary evidence rather than definitive clinical conclusions. Based on these results, and in combination with the efficacy performance and potential mechanisms of different OCPMs, the following stratified clinical recommendations can be proposed:

#### 4.3.1 Preferred options

Some OCPMs demonstrated relatively consistent advantages across multiple outcomes and may be considered as preferred options. For example, WFCT, MLP, and YWG showed notable effects in improving gastric mucosal inflammation, promoting glandular repair, and enhancing overall clinical efficacy, and may be suitable for patients with marked mucosal atrophy, persistent inflammation, and a higher risk of precancerous lesions. In addition, JHWKC and BLWTG showed advantages in improving *H. pylori* eradication and infection-related inflammation, and may be more suitable for *H. pylori*-positive patients with active inflammation. These agents act synergistically on the “*H. pylori*–inflammation–mucosal injury” axis, showing consistency between mechanistic rationale and statistical results.

#### 4.3.2 Pathology-oriented treatment selection

Some OCPMs exhibit advantages in specific outcomes or pathological processes and may be selected according to the patient’s primary clinical condition. For patients with impaired mucosal repair, YWG or KFXL may be preferred, as they have advantages in promoting mucosal repair, improving pepsinogen levels, and restoring glandular function. For patients with gastrointestinal motility disorders, such as postprandial fullness and dyspepsia, ZZKZC or WSG may be beneficial due to their regulatory effects on gastrointestinal hormones and gastric emptying. For patients with prominent inflammation or immune imbalance, WFCT and MLP may exert more significant effects through modulation of inflammatory signaling pathways.

#### 4.3.3 Combination therapy and individualized treatment

For patients with multiple pathological features (e.g., *H. pylori* infection combined with mucosal atrophy and motility disorders), combination strategies involving OCPMs with different mechanisms may be considered under the guidance of TCM syndrome differentiation. For example, combining agents with anti-*H. pylori* effects and those promoting mucosal repair or regulating motility may achieve synergistic effects across multiple pathological processes.

However, attention should be paid to avoiding overlapping components and potential drug interactions. Currently, high-quality evidence supporting combination strategies is still limited, and further studies are needed to verify their safety and efficacy.

Overall, the findings suggest that OCPMs combined with quadruple therapy may provide advantages over quadruple therapy alone in the treatment of CAG. The differences in efficacy among interventions may be attributed to their distinct mechanisms targeting different pathological processes, including *H. pylori* infection, inflammation, mucosal repair, and gastrointestinal function regulation.

However, given the generally low quality of evidence and the lack of direct comparative studies and TCM syndrome stratification, clinical application should be individualized based on patient conditions, syndrome differentiation, and drug availability. Future multicenter, large-scale, well-designed randomized controlled trials, together with mechanistic studies, are needed to further clarify the optimal use of OCPMs and support precision treatment strategies for CAG.

Given the low to very low certainty of the evidence, these clinical implications should be interpreted as exploratory and hypothesis-generating rather than definitive practice recommendations.

### 4.4 Limitations

#### 4.4.1 Methodological limitations of included studies

The overall methodological quality of the included randomized controlled trials was limited. Some studies reported using an “envelope method” for randomization but failed to describe essential features such as opacity, sequential numbering, or tamper-proof seals. Without such details, the adequacy of allocation concealment cannot be verified, and selection bias remains possible. In addition, no study reported blinding of participants, personnel, or outcome assessors. These methodological shortcomings may systematically inflate treatment effects, particularly for subjective outcomes, and substantially reduce the confidence in the findings. In addition, the generally small sample sizes may reduce statistical power and affect the reliability of the results. Collectively, these methodological defects may inflate the estimated treatment effects and reduce the reliability of evidence interpretation.

#### 4.4.2 Heterogeneity and inconsistency

Differences in diagnostic criteria, treatment duration, dosage regimens, and outcome measurements across studies may contribute to clinical heterogeneity. Moreover, some outcomes relied heavily on indirect comparisons, which may affect the consistency assumption and reduce the robustness of the network meta-analysis results. Meanwhile, variable components of quadruple therapy and wide time span of treatment courses further increased clinical heterogeneity, challenging the transitivity assumption of network meta-analysis.

#### 4.4.3 Variability in dosage and treatment duration

The dosage, frequency, and duration of the same OCPMs varied considerably across the included studies, with treatment courses ranging from 2 weeks to 6 months. A major limitation of this network meta-analysis is the inability to perform subgroup or meta-regression analyses based on treatment duration. Treatment duration is a known effect modifier for *H. pylori* eradication and gastric mucosal healing, and its omission from formal analysis significantly limits the interpretability of our findings. The primary reason for this omission is not oversight but data limitation. The included primary studies did not report outcome measures stratified by treatment duration, nor did they provide individual patient-level data. When we attempted to group studies by their reported fixed treatment windows, the number of trials per OCPM within each duration category was too small to generate stable effect estimates or to perform reliable statistical testing. Consequently, the pooled effect estimates and SUCRA rankings presented in this study represent averages across heterogeneous treatment durations. The potential modifying effect of treatment duration on the observed results remains unknown. Readers should interpret all findings with particular caution, and future research should standardize treatment duration or incorporate duration-stratified analyses from the outset.

#### 4.4.4 Structural limitations of the evidence network

Almost all outcome evidence networks were star-shaped without closed loops, and no direct head-to-head comparisons between different OCPMs were available. Indirect comparisons were only conducted via the common comparator of quadruple therapy, which precluded formal inconsistency assessment and lowered the stability of SUCRA ranking results. Accordingly, all rankings should be interpreted merely as exploratory trends rather than definitive efficacy hierarchy.

#### 4.4.5 Transitivity and clinical heterogeneity

The validity of network meta-analysis depends on the assumption of transitivity, which requires comparability across studies. Although all included trials involved patients with CAG and used quadruple therapy as the background treatment, differences in *H. pylori* infection status, disease duration, and severity of atrophy or intestinal metaplasia may act as effect modifiers. In addition, the specific components of quadruple therapy varied across studies, including different types of proton pump inhibitors, antibiotic combinations, and treatment durations ranging from 7 to 14 days. While this variation reflects real-world clinical diversity, it may theoretically threaten the transitivity assumption. However, there is no evidence of systematic pairing between specific quadruple therapy regimens and particular Chinese patent medicines, which would have biased indirect comparisons. Therefore, the findings should be interpreted with caution.

#### 4.4.6 Surrogate outcomes and lack of long-term endpoints

All outcomes assessed in this study were surrogate biomarkers including inflammatory factors, gastrointestinal hormones and pepsinogen indices. No trials reported patient-centered clinical outcomes, histological improvement of gastric atrophy and intestinal metaplasia, or long-term endpoints such as disease progression and gastric cancer occurrence. Thus, biomarker improvement cannot be equated with clinically meaningful reversal of precancerous lesions.

#### 4.4.7 Lack of TCM syndrome differentiation

Although all included studies used OCPMs, most did not incorporate TCM syndrome differentiation. According to TCM principles, OCPMs should be prescribed based on specific syndromes, and treatment responses may vary significantly among patients with different syndromes. The use of Western diagnostic criteria alone may result in heterogeneous populations (“same disease, different syndromes”), potentially obscuring true treatment effects.

#### 4.4.8 Publication bias and small-sample effects

Most included studies were single-center and small-sample trials, and positive results are more likely to be published, which may lead to publication bias. Indeed, Egger’s test indicated potential publication bias for several outcomes, including *H. pylori* eradication rate, PGII, and IL-6 (*p* < 0.05). In addition, many outcomes (e.g., GAS, MTL, PGI/PGII, CRP, IL-8, and IL-1β) were based on a limited number of studies. Under such conditions, Egger’s test has low statistical power, and small-sample effects may affect the stability of the results. Therefore, publication bias cannot be reliably assessed for these outcomes, and the findings should be interpreted with caution. For most outcomes with limited study numbers, the statistical power of Egger’s test was insufficient; therefore, potential publication bias could not be reliably excluded.

### 4.5 Future directions

Future research should focus on improving both the quality of clinical evidence and the depth of mechanistic understanding regarding OCPMs in the treatment of chronic atrophic gastritis (CAG). First, there is an urgent need for well-designed, multicenter, large-sample randomized controlled trials with standardized diagnostic criteria, intervention protocols, and outcome measurements to enhance the reliability and comparability of clinical evidence. In particular, head-to-head trials directly comparing different OCPMs are warranted to validate the relative efficacy suggested by network meta-analysis.

Second, future studies should incorporate long-term follow-up endpoints, such as histological improvement of gastric mucosal atrophy and intestinal metaplasia, as well as the incidence of gastric cancer, in order to better evaluate the disease-modifying effects of OCPMs beyond short-term symptom relief and biochemical improvements.

Third, integration of traditional Chinese medicine syndrome differentiation into clinical trial design is essential. Stratified analyses based on TCM patterns may help to identify patient subgroups that benefit most from specific OCPMs, thereby facilitating more precise and individualized treatment strategies.

Fourth, advances in multi-omics technologies, including transcriptomics, proteomics, and metabolomics, should be leveraged to systematically elucidate the multi-target and multi-pathway mechanisms of OCPMs. Such approaches may help to clarify how different interventions modulate key pathological axes in CAG, including *Helicobacter pylori*-related injury, chronic inflammation, mucosal repair, and precancerous transformation.

Finally, future research should also explore optimal combination strategies between OCPMs and conventional therapies, as well as among different OCPMs with complementary mechanisms, while carefully evaluating potential drug interactions and safety profiles.

## 5 Conclusion

In summary, this NMA systematically explored the comparative efficacy of 10 OCPMs combined with QT for CAG. Combined regimens generally exhibited potential advantages over quadruple therapy alone in terms of *H. pylori* eradication, inflammatory modulation and improvement of gastric mucosal functional biomarkers. Different patent medicines displayed distinct rank trends across multiple outcome indicators, which may be related to their multi-target regulatory mechanisms acting on the pathological axis of *H. pylori* infection-chronic inflammation-gastric mucosal injury-precancerous progression.

Nevertheless, several inherent limitations should be explicitly acknowledged. First, nearly all evidence networks adopted a star structure without closed loops, and all comparisons relied on indirect evidence without head-to-head randomized controlled trials, weakening the robustness of SUCRA rankings. Second, the included primary studies suffered from suboptimal methodological quality, inadequate allocation concealment, absence of blinding, and single-center small-sample design. Third, substantial heterogeneity existed in the components of quadruple therapy, treatment duration and diagnostic criteria across trials. Fourth, all evaluated endpoints were surrogate biochemical biomarkers; no long-term hard outcomes such as histological reversal of atrophy/intestinal metaplasia or gastric cancer incidence were reported. Fifth, subgroup analysis and meta-regression could not be performed due to insufficient raw data, leaving the modifying effects of treatment course, dosage and TCM syndrome types unclarified.

Overall, the certainty of current evidence is rated as low to very low. The results presented herein are only exploratory and hypothesis-generating, and cannot be used as definitive clinical recommendations. Rational clinical application should adhere to individualized syndrome differentiation. Future well-designed, multicenter, large-sample randomized controlled trials together with mechanism exploration are warranted to validate these preliminary findings.

## Data Availability

The original contributions presented in the study are included in the article/Supplementary material, further inquiries can be directed to the corresponding author.

## References

[1] Wu GB Cheng XL Yu X Zhang AB Liu M Chen ZF. Research Progress on the relationship between *Helicobacter Pylori* associated chronic atrophic gastritis and gastric Cancer. Med J Peking Union Medical College Hospital. (2023) 14:339–45. doi: 10.12290/xhyxzz.2022-0374

[2] Xia C Yang CP Sun ZH Dong MZ Zhang JL Yang J . Research Progress on the correlation between gastric xanthoma and early gastric Cancer and precancerous lesions. Diagnosi. (2025) 15:148–53. doi: 10.12677/md.2025.152020

[3] Li Y Jiang F Wu CY Leung WK. Prevalence and temporal trend of gastric preneoplastic lesions in Asia: a systematic review with meta-analysis. United European Gastroenterol J. (2024) 12:139–51. doi: 10.1002/ueg2.12507, 38084663 PMC10859711

[4] Wang NF Chu HK Huang SM Xu YJ Fang JN. Clinical characteristics of chronic atrophic gastritis patients in Heilongjiang province. Chin J Public Health. (2017) 33:1109–11. doi: 10.11847/zgggws2017-33-07-20

[5] Digestive System Diseases Professional Committee of Chinese Association of Integrative Medicine. Expert consensus on integrated traditional Chinese and Western medicine diagnosis and treatment of chronic atrophic gastritis (2025). Chin J Integr Trad West Med Dig. (2025) 33:230–41. doi: 10.3969/jissn1671-038X20250.04

[6] Zhang J Shang YX Yang QR Lei YY Chen H Li CL . Current status of outcome indicators in randomized controlled trials of traditional Chinese medicine for treating chronic atrophic gastritis. Chin J Exp Tradit Med Formulae. (2024) 30:193–202. doi: 10.13422/j.cnki.syfjx.20231714

[7] Fu YQ Han X Liang S Fb L. Advances in the pathogenesis of chronic atrophic gastritis. Chin J Integr Trad West Med Dig. (2023) 31:479–84. doi: 10.3969/j.issn.1671-038X.2023.06.16

[8] Yuan HW Wang CH. Combined detection of pepsinogen and gastrin in the diagnosis of chronic atrophic gastritis. Chin J Integr Trad West Med Dig. (2014) 22:33–5. doi: 10.3969/j.issn1671-038X2014.01.012

[9] Zhao JF Wu Y Wang XY Liang LW Gan Y. Clinical effects of Yangwei granules combined with quadruple therapy on *helicobacter pylori* positive chronic atrophic gastritis and its influence on gastrin, endothelin and pepsinogens. Chin J Integr Trad West Med Dig. (2018) 26:640–4. doi: 10.3969/j.issn.1671-038X.2018.08.03

[10] Zhang LL Geng L Lin XR Dang H. Clinical study of Yangwei granules combined with quadruple therapy in the treatment of chronic atrophic gastritis with positive Hp. Chin Med Herald. (2019) 16:152–5.

[11] Zhu TY Kong HW Yin QH Jiang GF. Observation of the efficacy of Yangwei granule in the treatment of chronic atrophic gastritis with Hp infection. China Continuing Medical Education. (2020) 12:171–4. doi: 10.3969/j.issn.1674-9308.2020.36.049

[12] Xiong HP Xie WX. Clinical study on Yangwei granules combined with quadruple therapy for chronic atrophic gastritis complicated with *Helicobacter pylori* infection [J]. The Primary Medical Forum. (2023) 27:130–2. doi: 10.19435/j.1672-1721.2023.25.042

[13] Liu HG. Observation on the efficacy of Yangwei granules as adjuvant therapy in *Helicobacter pylori* (Hp)-positive chronic atrophic gastritis (CAG). Womens Health. (2023) 20:169–70.

[14] Jia CJ. Effect observation of Yangwei granules as adjuvant therapy for *Helicobacter pylori*-positive chronic atrophic gastritis. Special Health. (2023) 3:115–6. doi: 10.3969/j.issn.2095-6851.2023.03.061

[15] Liang Q Lan P Yang C Zhu WJ Kong QY. Clinical study on treatment of chronic atrophic gastritis with Weisu granule combined with quadruple therapy. Int J Traditional Chinese Medicine. (2019) 41:688–91. doi: 10.3760/cma.j.issn.1673-4246.2019.07.004

[16] Qi HL. Effect of Weisu granules combined with quadruple therapy in the treat-ment of Hp positive chronic atrophic gastritis of the spleen and stomachwith gas stagnation. China Modern Med. (2021) 28:43–9. doi: 10.3969/j.issn.1674-4721.2021.16.012

[17] Huang MX Li ZJ Lin PL Li HX Zhong HX. Efficacy of Weisu granules combined with quadruple therapy in the treatment of HelicobacterPylori positive chronic atrophic gastritis. Evaluation and analysis of drug-use in hospitals of China. (2021) 21:281–4. doi: 10.14009/j.issn.1672-2124.2021.03.006

[18] Wang M Wu LL Zhou Q Wu C Shi H. Effect of Weisu granule combined with quadruple therapy on serum gastrointestinal hormone and gastric mucosal COX-2 and NF-κB in patients with Hp positive chronic atrophic gastritis. Progress in modern. Biomedicine. (2022) 22:1856–1859,1864. doi: 10.13241/j.cnki.pmb.2022.10.012

[19] Jin LN. The value of Weisu granules combined with quadruple therapy in the treatment of chronic atrophic gastritis patients with *helicobacter pylori* positive. Shanghai. Pharm J. (2023) 44:28–50. doi: 10.3969/j.issn.1006-1533.2023.20.008

[20] Zhang XX. Analysis of inflammatory factors of Weisu granules combined with quadruple therapy in chronic atrophic gastritis with Hp infection. Med Health. (2024) 12:134–8.

[21] Chen XJ. Observation on the curative effect of Weisu granules in the treatment of chronic atrophic Gastritisand its effect on serum inflammatory factors and gastrointestinal hormones. Journal of Med Information. (2025) 38:113–21. doi: 10.3969/j.issn.1006-1959.2025.16.023

[22] Ma XX. Clinical efficacy of Weifuchun combined with quadruple therapy in the treatment of chronic atrophic gastritis caused by *Helicobacter pylori* infection. Contemporary Medical Symposium. (2020) 18:150–1. doi: 10.3969/j.issn.2095-7629.2020.15.109

[23] Wang WG. Effects of Weifuchun tablets and esomeprazole on the levels of IL-6, IL-8 and TNF-α in patients with chronic atrophic gastritis. World Journal of Complex Medicine. (2020) 6:178–80. doi: 10.11966/j.issn.2095-994X.2020.06.06.59

[24] Munila MMT Feng Y Gao F. Analysis of therapeutic efficiency and adverse reactions of quadruple therapy combined with Weifuchun in the treatment of chronic atrophic gastritis. Women's Health Res China and Foreign Countries. (2021) 20:113–4.

[25] Wang HX. Efficacy of Weifuchun tablet combined with quadri-combination therapy of rabeprazole in the treatment of chronic atrophic gastritis patients complicated with Hp infection. Acta Medicinae Sinica. (2021) 34:22–6. doi: 10.19296/j.cnki.1008-2409.2021-03-006

[26] Hu JY. The effect of Weifuchun combined with quadruple therapy on gastrointestinal hormones and immune function in patients with chronic atrophic gastritis. J Med Theor Prac. (2021) 34:956–8. doi: 10.19381/j.issn.1001-7585.2021.06.023

[27] Wang H Xie XH Bi YZ Yu L. Clinical study on Weifuchun combined with bismuth quadruple therapy for atrophic gastritis complicated with Hp infection. New Chinese Med. (2024) 56:85–9. doi: 10.13457/j.cnki.jncm.2024.06.016

[28] Xiao ZQ. Clinical observation of Morodan combined with quadruple therapy in the treatment of *Helicobacter Pylori*-positive chronic atrophic gastritis. China's Naturopathy. (2019) 27:67–8. doi: 10.19621/j.cnki.11-3555/r.2019.1237

[29] Wang L. Control effect of Moluodan combined with rabeprazole quadruple therapy on Hp-positive chronic atrophic gastritis. Chin J of public Health Eng. (2020) 19:623–4.

[30] Chen CY. Effect of Moluodan combined with quadruple therapy in the treatment of *Helicobacter Pylori*-related chronic atrophic gastritis. Chinese and Foreign Medical Res. (2024) 22:131–5. doi: 10.14033/j.cnki.cfmr.2024.20.033

[31] Li HL. Clinical study on Moluo pellets combined with quadruple therapy for *Helicobacter Pylori*-positive chronic atrophic gastritis. New Chinese medicine. (2024) 56:33–8. doi: 10.13457/j.cnki.jncm.2024.03.007

[32] Shang PJ Xia YX Liu XZ. Efficacy of Morodan combined with bismuth-containing quadruple therapy for *Helicobacter Pylori*-positive chronic atrophic gastritis. Chinese Journal of Rational Drug Use Exploration. (2025) 22:93–8. doi: 10.3969/j.issn.2096-3327.2025.05.015

[33] Wu QF Yang XW Cheng C. The application effect of Moluodan + modified quadruple sequential therapy in Hp-positive chronic atrophic gastritis. Med Innovation of China. (2025) 22:9–13. doi: 10.3969/j.issn.1674-4985.2025.12.003

[34] Li D Liu DQ Qi WJ Du SJ. Clinical observation of Zhizhu Kuanzhong capsules combined with western medicine in the treatment of *Helicobacter pylori*-positive chronic atrophic gastritis. Chinese Journal of Clinicians. (2018) 46:1117–20. doi: 10.3969/j.issn.2095-8552.2018.09.038

[35] Li D Yu YQ Liu DQ. Clinical effect of Zhizhu Kuanzhong capsules combined with probiotics on *Helicobacter pylori*-positive elderly patients with chronic atrophic gastritis. Chin J Microeco. (2019) 31:429–38. doi: 10.13381/j.cnki.cjm.201904011

[36] Li JH Cen CC. Clinical observation of Zhizhu Kuanzhong capsules combined with Western medicine in the treatment of *Helicobacter Pylori*-positive chronic atrophic gastritis. Inner Mongolia J Traditional Chinese Med. (2020) 39:65–6. doi: 10.16040/j.cnki.cn15-1101.2020.11.040

[37] Liu GL Gu XR. Observation on the effect of Zhizhu Kuanzhong capsules combined with quadruple therapy in the treatment of elderly patients with Hp-positive chronic atrophic gastritis. Women's Health Research. (2022) 8:121.

[38] Guan JF Mi CF. Effect of quadruple therapy combined with Jinghua Weikang in the treatment of Hp-positive chronic atrophic gastritis. Clinical Medical Engineering. (2022) 29:485–6. doi: 10.3969/j.issn.1674-4659.2022.04.0485

[39] Ni H Guan XH. Effect of quadruple therapy combined with Jinghua Weikang in the treatment of Hp-positive chronic atrophic gastritis. Journal of Beihua University (Natural Science). (2023) 24:626–9. doi: 10.11713/j.issn.1009-4822.2023.05.012

[40] Li Y Zhao T. Effect of standard quadruple combined with Jinghua Weikang capsule on the levels of serum gastrin 17 and PG, and immune function in chronic atrophic gastritis patients with positive Hp. J Bengbu Med Coll. (2023) 48:222–5. doi: 10.13898/j.cnki.issn.1000-2200.2023.02.020

[41] Li WH Hou J Ma XZ. Observation on the efficacy of Jinghua Weikang capsules as adjuvant therapy in the treatment of chronic atrophic gastritis. Journal of practical traditional Chinese medicine. (2025) 41:1567–9. doi: 10.3969/j.issn.1004-2814.2025.08.019

[42] Lu XP Huang J. Treatment of 41 cases of chronic atrophic gastritis with Qi stagnation and blood stasis syndrome by Biling Weitong granules. Jiangxi Journal of Traditional Chinese Medicine. (2020) 51:49–51.

[43] Hong SS Huang JH Xu SJ. Effect of Biling Weitong granules combined with quadruple therapy on chronic atrophic gastritis with positive *Helicobacter pylori* infection. China Medical Herald. (2021) 18:122–5.

[44] Su WX Liu Q Du BJ Gao ZW He D Zhao JJ . Biling Weitong granules combined with quadruple therapy in the treatment of chronic atrophic gastritis complicated with *Helicobacter pylori* infection: efficacy and its effect on serum levels of inflammatory factors, EGF and TGF-β1. Modern Journal of Integrated Traditional Chinese and Western Medicine. (2023) 32:692–5. doi: 10.3969/j.issn.1008-8849.2023.05.022

[45] Gong Y Fei Y Mao LL Ren J Chen H Wang DR. Clinical efficacy of Biling Weitong granules combined with quadruple therapy in treating *Helicobacter Pylori*-positive chronic atrophic gastritis patients and its protective effect on gastric mucosa. World Journal of Integrated Traditional and Western Medicine. (2024) 19:1832–8. doi: 10.13935/j.cnki.sjzx.240923

[46] Sun JC Huang SL Liu TT Pan YF. Efficacy of Wumei pills combined with anti-Hp quadruple eradication therapy for the treatment of Hp-infected chronic atrophic gastritis of cold-heat concurrence type and their effect on Hp eradication rate and serum levels of IL-1β and IL-6. J Guangzhou University of Traditional Chinese Medicine. (2023) 40:1916–22. doi: 10.13359/j.cnki.gzxbtcm.2023.08.010

[47] Mao L Zhou LP Li CL. Clinical efficacy of Wumei pill for *helicobacter pylori* positive chronic atrophic gastritis of cold and heat miscellaneous type. Hebei J Tradit Chin Med. (2024) 46:1977–85. doi: 10.3969/j.issn.1002-2619.2024.12.009

[48] Zhao XQ Hu XY. Effect of Wumei pills combined with quadruple therapy on Hp eradication rate and inflammatory indexes in patients with Hp-positive chronic atrophic gastritis. Health Friend. (2025) 13:49–51. doi: 10.3969/j.issn.1002-8714.2025.13.017

[49] Huang JQ. Clinical Observation of Sanqi Shengweipill Combined with Quadruple Therapyin the Treatment of Chronic Atrophicgastritis Caused by Hp Infection. Enshi City: Hubei Minzu University (2023).

[50] Zeng ZX. Effect analysis of clinical efficacy of Sanqi Shengwei pills combined with quadruple therapy in the treatment of chronic atrophic gastritis with Hp infection. Med Health. (2024) 8:078–81.

[51] Huang JQ Deng H Lv PH Wang W Bi XJ Liu P . Clinical observation on the efficacy of Sanqi Shengwei pills combined with bismuth-containing quadruple therapy in the treatment of chronic atrophic gastritis (Yin deficiency and blood stasis syndrome) with Hp infection proceedings of the 2^nd^ Chinese Journal of Histochemistry and Cytochemistry Clinical Medicine Symposium of Chinese Journal of Histochemistry and Cytochemistry in 2025. Beijing (2025).

[52] Sheng LH. Clinical observation on the effect of Kangfuxin liquid combined with killing *Helicobacter pylori* on chronic atrophic gastritis. Chinese Community Doctors. (2019) 35:62. doi: 10.3969/j.issn.1007-614x.2019.11.062

[53] Zhao WQ. Clinical study on Kangfuxin liquid combined with quadruple therapy for *Helicobacter Pylori* positive chronic atrophic gastritis. J New Chinese Medicine. (2020) 52:59–62. doi: 10.13457/j.cnki.jncm.2020.04.018

[54] Nikolakopoulou A Higgins JPT Papakonstantinou T Chaimani A Del Giovane C Egger M . CINeMA: an approach for assessing confidence in the results of a network meta-analysis. PLoS Med. (2020) 17:e1003082. doi: 10.1371/journal.pmed.1003082, 32243458 PMC7122720

[55] Papakonstantinou T Nikolakopoulou A Higgins JPT Egger M Salanti G. CINeMA: software for semi automated assessment of the confidence in the results of network meta-analysis. Campbell Syst Rev. (2020) 16:e1080. doi: 10.1002/cl2.1080, 37131978 PMC8356302

[56] Wang Q Wang YH Lai HH Wang Q Ding GW Tian JH . Rating the certainty of evidence from network meta-analysis: an introduction to CINeMA. Chin J Evid Based Med. (2020) 20:1111–6. doi: 10.7507/1672-2531.202002172

[57] Fang JY Du YQ Liu WZ Xiao YL Chen WC Ren JL . Guidelines for diagnosis and treatment of chronic gastritis in China (2022, Shanghai). Chin J Gastroenterol. (2023) 28:149–80. doi: 10.3969/j.issn.1008-7125.2023.03.004

[58] Kuang W Xu J Xu F Huang W Majid M Shi H . Current study of pathogen etic mechanisms and therapeutics of chronic atrophic gastritis: a comprehensive review. Front Cell Dev Biol. (2024) 12:1513426. doi: 10.3389/fcell.2024.1513426, 39720008 PMC11666564

[59] Xu Q Liu M Meng R Zhao Q Men X Lan Y . Therapeutic effects and potential mechanisms of endoscopic submucosal injection of mesenchymal stem cells on chronic atrophic gastritis. Sci Rep. (2023) 13:20745. doi: 10.1038/s41598-023-48088-3, 38007523 PMC10676420

[60] Zha T Ding Y Xu X Zhang Y Guo J Ge H . The oral-gut axis in chronic atrophic gastritis: current perspectives and integrated strategies. Front Immunol. (2026) 16:1699501. doi: 10.3389/fimmu.2025.1699501, 41583463 PMC12823898

[61] Lin HC Zhou M. Research progress on the mechanism of traditional Chinese medicine in the treatment of chronic atrophic gastritis. China modern medicine. (2024) 31:185–94.

[62] Suo ZQ Li HZ JL MA Zhao SM. Research progress on experimental mechanism of traditional Chinese medicine treatment of chronicatrophicgastritis. Chin J Integr Trad West Med Dig. (2024) 32:627–38. doi: 10.3969/j.issn.1671-038X.2024.07.16

[63] Wei CL Chen L Luo B Chen JY. Research progress of mechanism about Jinghua Weikang capsule against Helicobacterpylori. Chin J Integr Trad West Med Dig. (2024) 32:730–4. doi: 10.3969/j.issn.1671-038X.2024.08.18

[64] Liu ZX Hou L. Research progress in the treatment of *Helicobacter pylori* infection with traditional Chinese medicine. Yaoxue QianYan Zazhi. (2024) 28:144–54. doi: 10.12173/j.issn.2097-4922.202404140

[65] Dai A Yao YL Zhou YQ. Efficacy of Bilin Weitong granules combined with lansoprazole quadruple therapy in patients with chronic gastritis complicated with *Helicobacter pylori* infection. Modern Digestion Intervention. (2020) 25:515–7.

[66] Liu YX Liu XZ Zheng LH. Research progress on traditional Chinese medicine regulating signaling pathways related to chronic atrophic gastritis. J Clin Personalized Med. (2024) 3:1554–60. doi: 10.12677/jcpm.2024.34223

[67] Yu QS Jiang GF Chen H He X. Observation on effect of Weifuchun combined with Teprenone on patients with chronic atrophic Gastritisand intestinal metaplasia. Progress in Pharmaceutical Sci. (2025) 49:478–80. doi: 10.20053/j.issn1001-5094.202503100100

[68] Chen YX Deng X Pan N Tan JX Liu FJY Huang XY. Research progress on clinical application of Weifuchun based on TCM "treating different diseases with the same method". J Tradit Chin Med. (2024) 13:563–71. doi: 10.12677/tcm.2024.133087

[69] Chen J He LL Gao Y Zhang K. Moluodan concentrated pill in the treatment of chronic atrophic gastritis through the TNF/PI3K/AKT signaling pathway. J Mathematical Med. (2024) 37:823–30. doi: 10.12173/j.issn.1004-4337.202408182

[70] China Association of Chinese Medicine. Project team of expert consensus on the clinical application of Yangwei granules in the treatment of chronic atrophic gastritis. Expert consensus on clinical application of Yangwei granules in treatment of chronic atrophic gastritis. Chin Arch Tradit Chin Med. (2024) 42:255–8. doi: 10.13193/j.issn.1673-7717.2024.09.049

[71] Huang SH Song JD Chu PC Xu BC. Clinical study on Yangwei granules for chronic atrophic gastritis with spleen Deficiency and Qi stagnation syndrome. J New Chinese Med. (2020) 52:70–2. doi: 10.13457/j.cnki.jncm.2020.12.021

[72] Zhao B Cai MQ Cui YR Liu JY. Efficacy and safety of Kangfuxin liquid in postoperative gastrointestinal endoscopy patients: a meta-analysis. Yaowu Liuxingbingxue Zazhi. (2024) 33:678–87. doi: 10.12173/j.issn.1005-0698.202402031

[73] Chen ML Geng FN Shen YM Zhag CL Que HF Guo WG . A multi-center, randomize-controlled clinical trial of Kangfuxin solution for patients with chronic skin ulcer. J Tradit Chin Med. (2019) 60:1308–11. doi: 10.13288/j.11-2166/r.2019.15.011

[74] Liu FF Wang JH Xu YP Mu H Yang YY. Effects of Zhizhu Kuanzhong capsules combined with mosapride on gastrointestinal hormones and gastric emptying in patients with functional dyspepsia of spleen deficiency and Qi stagnation. Clinical Res Pract. (2024) 9:125–8. doi: 10.19347/j.cnki.2096-1413.202409031

[75] Hou YF Li JC Zhang J. Effect of Zhizhu Kuanzhong capsules on patients with functional dyspepsia complicated with anxiety and depression. J Int Psychiatry. (2019) 46:721–3.

[76] Liang JR Zhou YQ Hu K Li CY Zou BC. Clinical effect of Weisu granules combined with domperidone on patients with functional dyspepsia with the syndrome of liver-stomach disharmony and effect of gastrointestinal hormone. Chin J Integr Trad West Med Dig. (2021) 29:272–5. doi: 10.3969/j.issn.1671-038X.2021.04.09

[77] Zhao JL Yu YX. Clinical observation on the efficacy of Weisu granules in the treatment of chronic gastritis. J Health Medicine, Chinese Science and Technology Journal Database. (2025) 5:050–3.

