# Supplementary figures and images for: Efficacy and safety of oral Chinese patent medicine combined with quadruple therapy for chronic atrophic gastritis: a systematic review and network meta-analysis
Source: Front Med (Lausanne). 2026 Jun 23;13:1859753. doi: 10.3389/fmed.2026.1859753 (PMC13337655; doi:10.3389/fmed.2026.1859753)

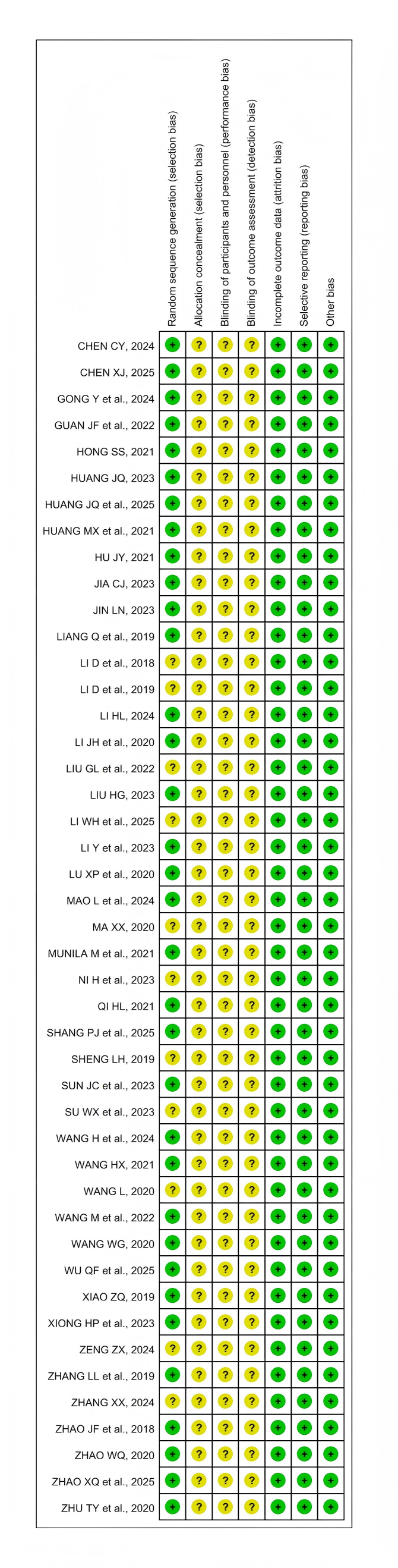

Supplement: Supplementary file 1 [file Image_1.TIF]

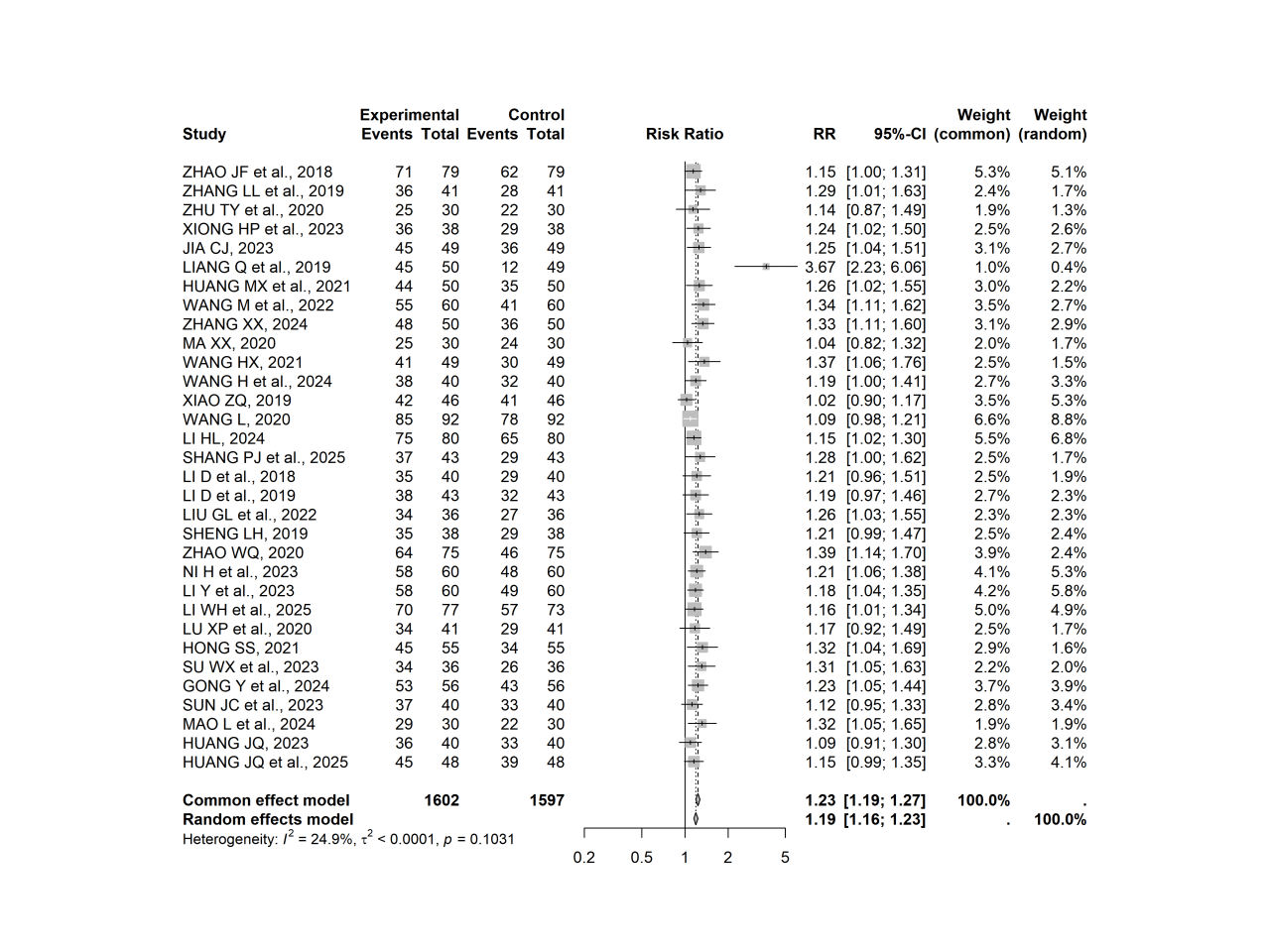

Supplement: Supplementary file 2 [file Image_2.PNG]

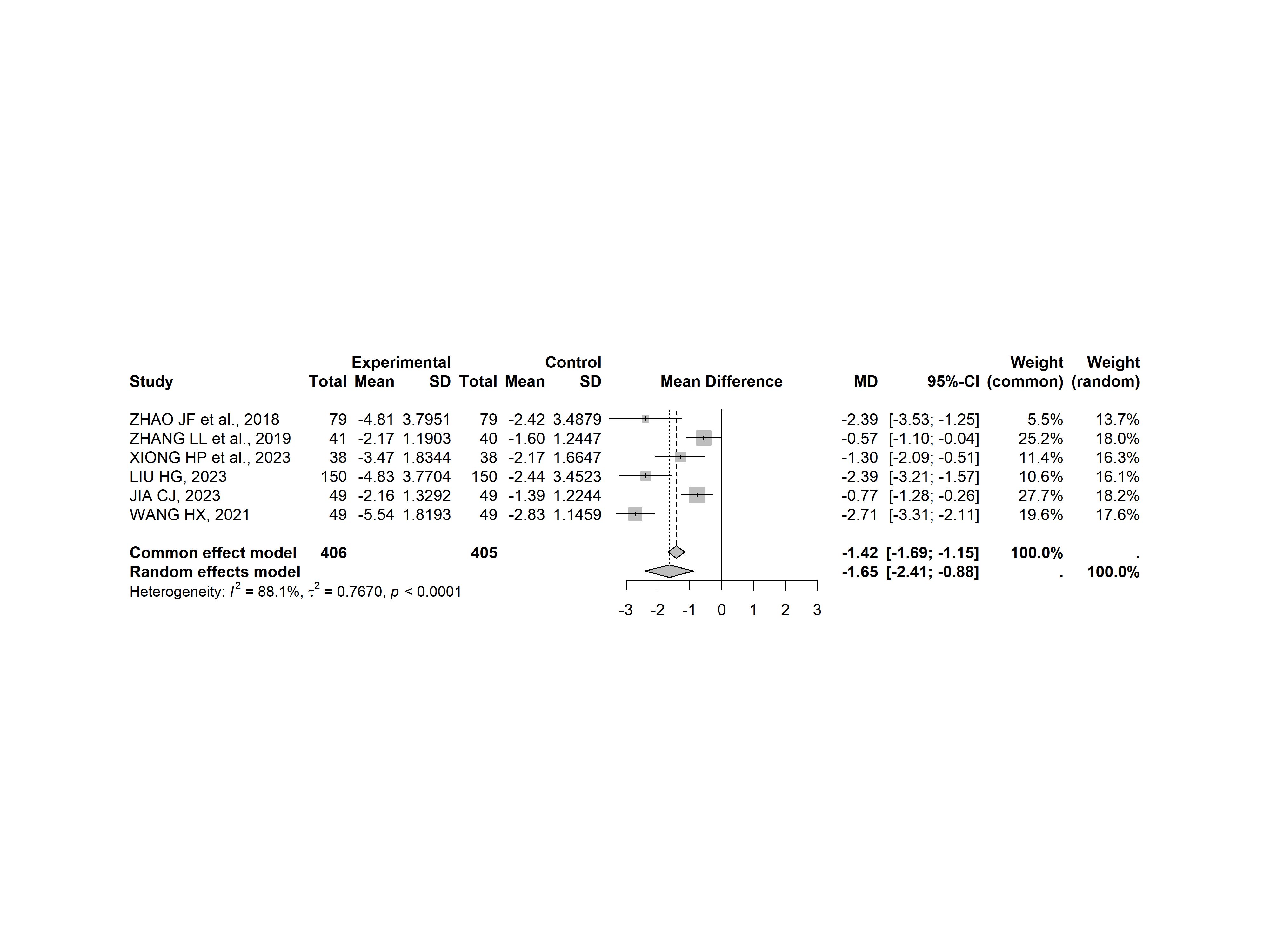

Supplement: Supplementary file 3 [file Image_3.PNG]

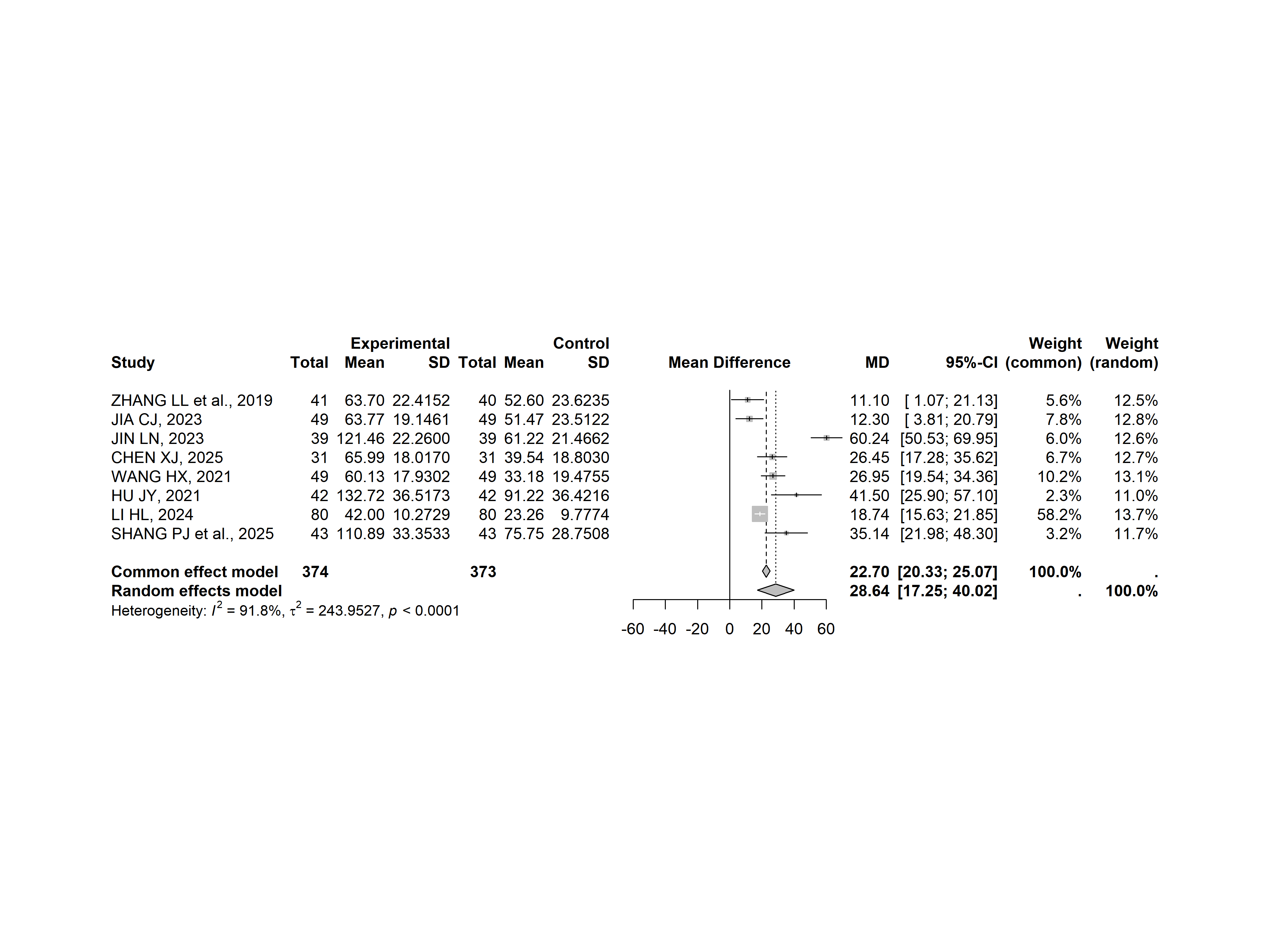

Supplement: Supplementary file 4 [file Image_4.PNG]

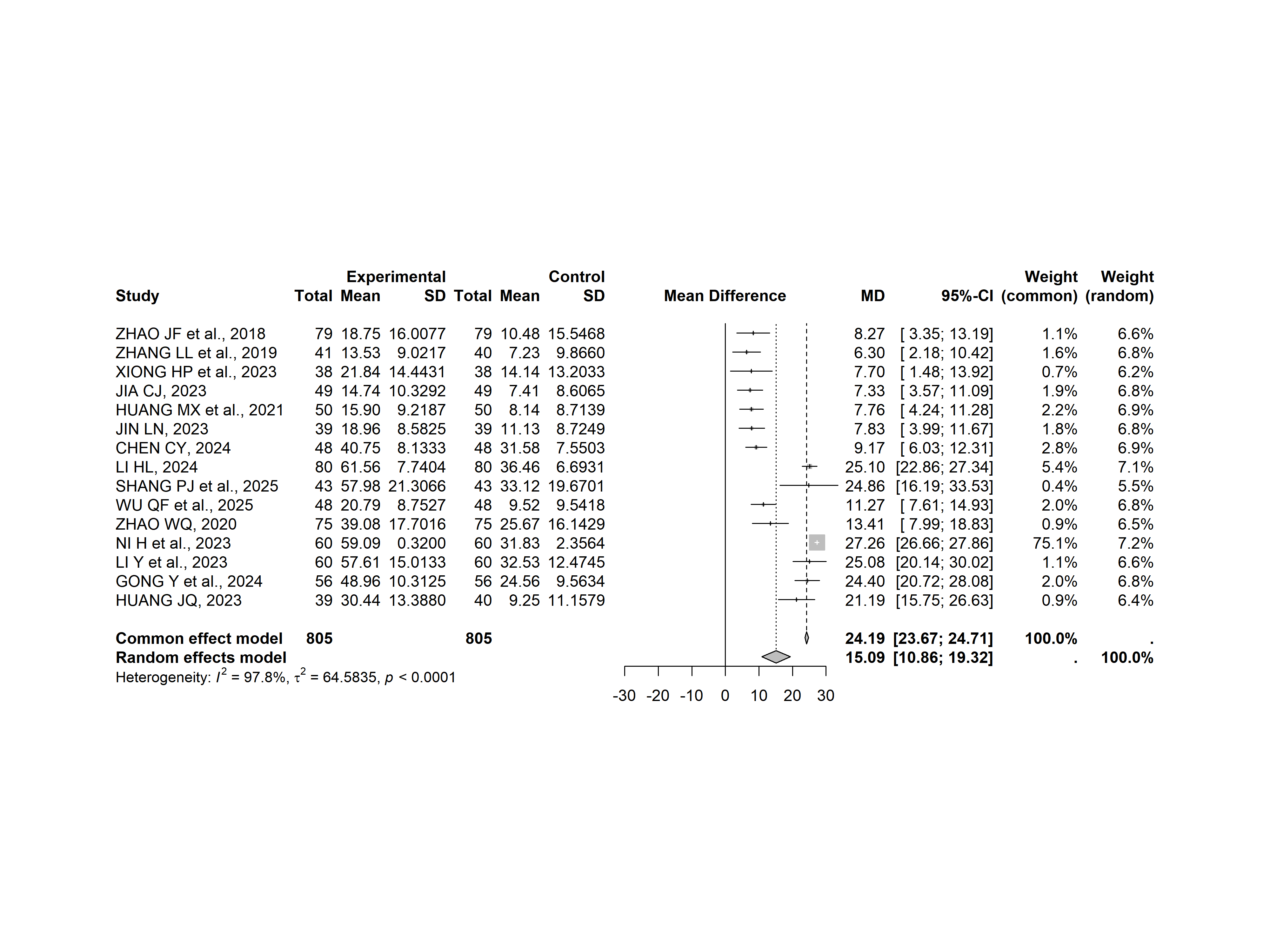

Supplement: Supplementary file 5 [file Image_5.PNG]

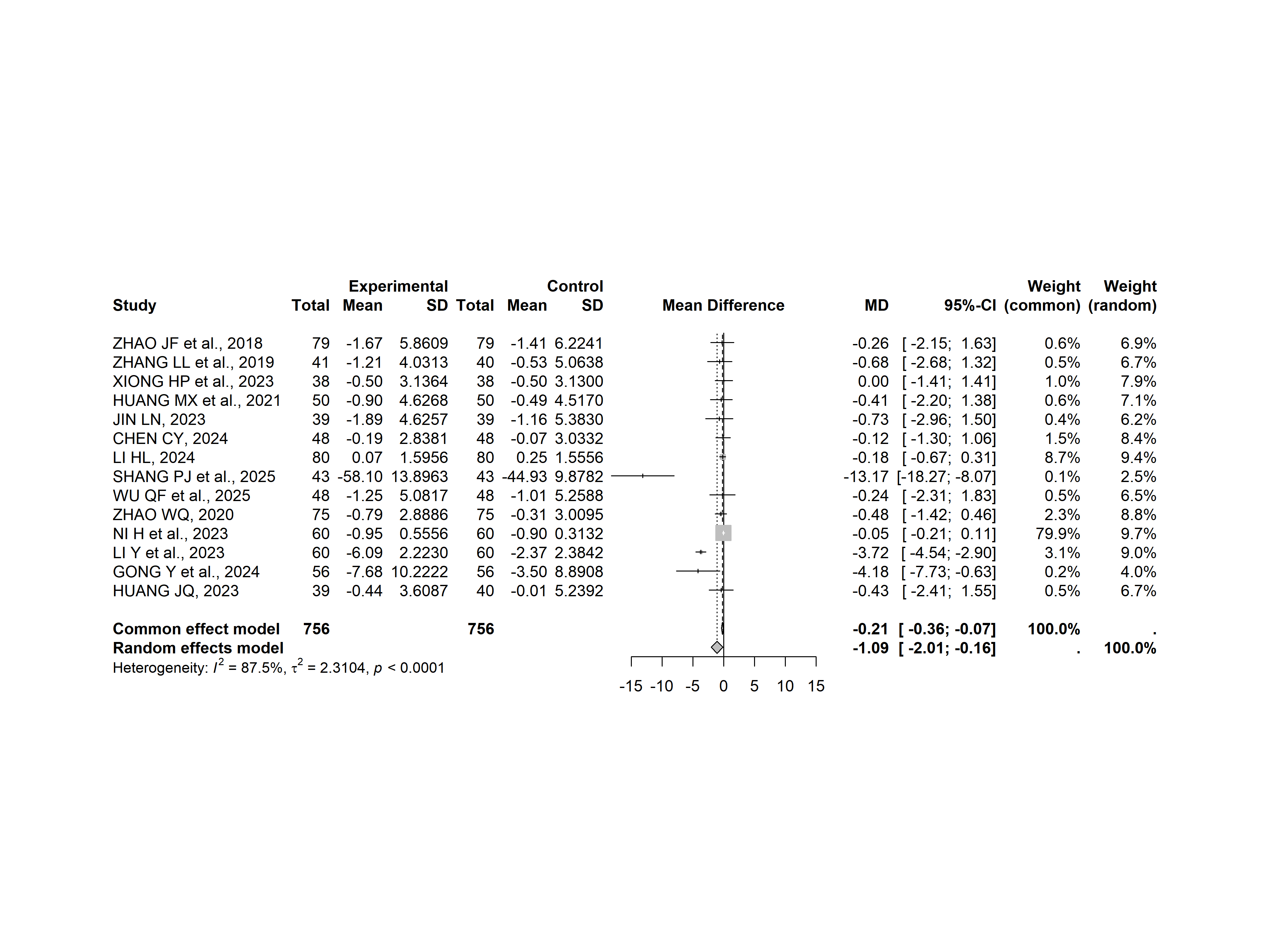

Supplement: Supplementary file 6 [file Image_6.PNG]

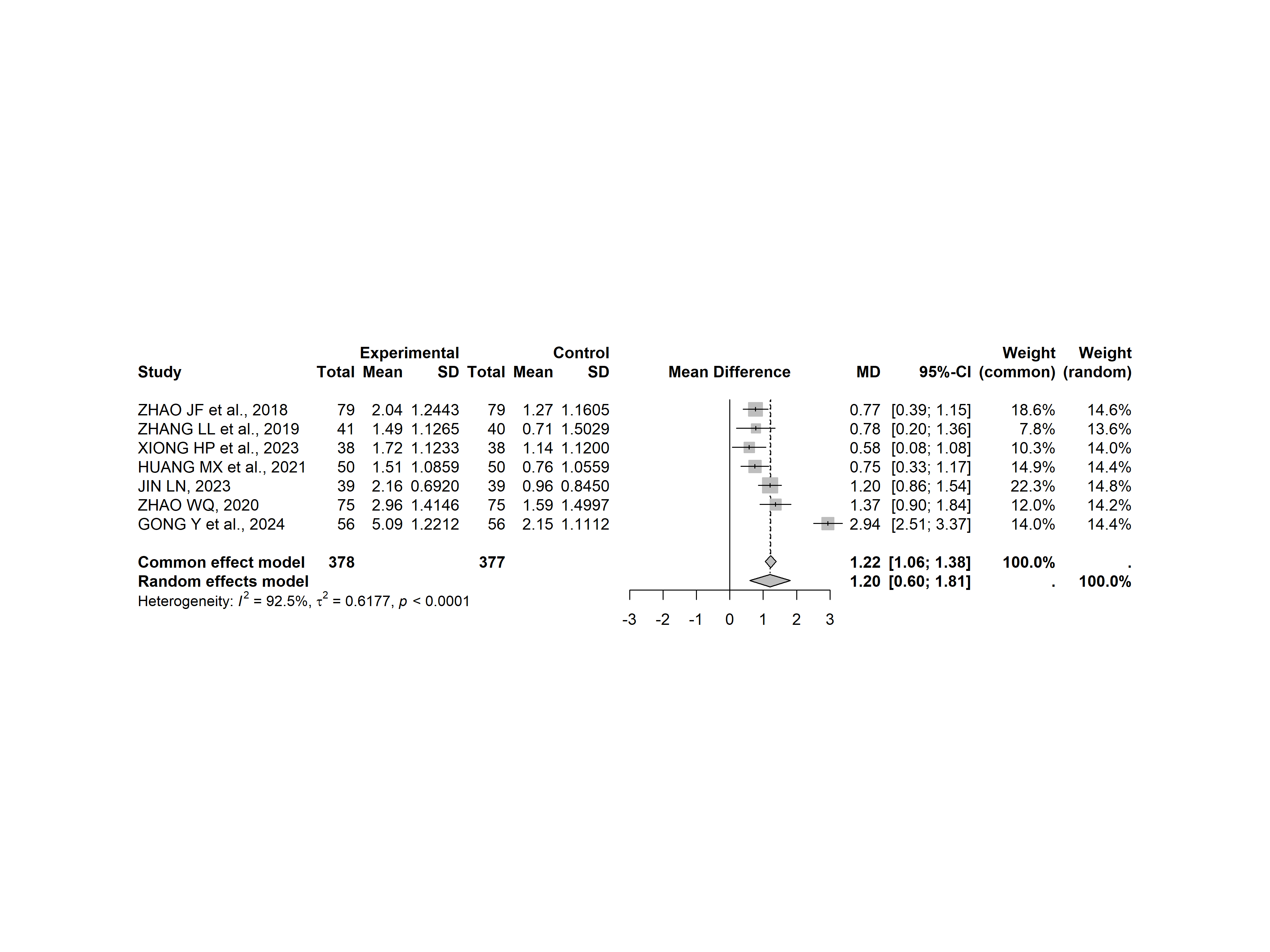

Supplement: Supplementary file 7 [file Image_7.PNG]

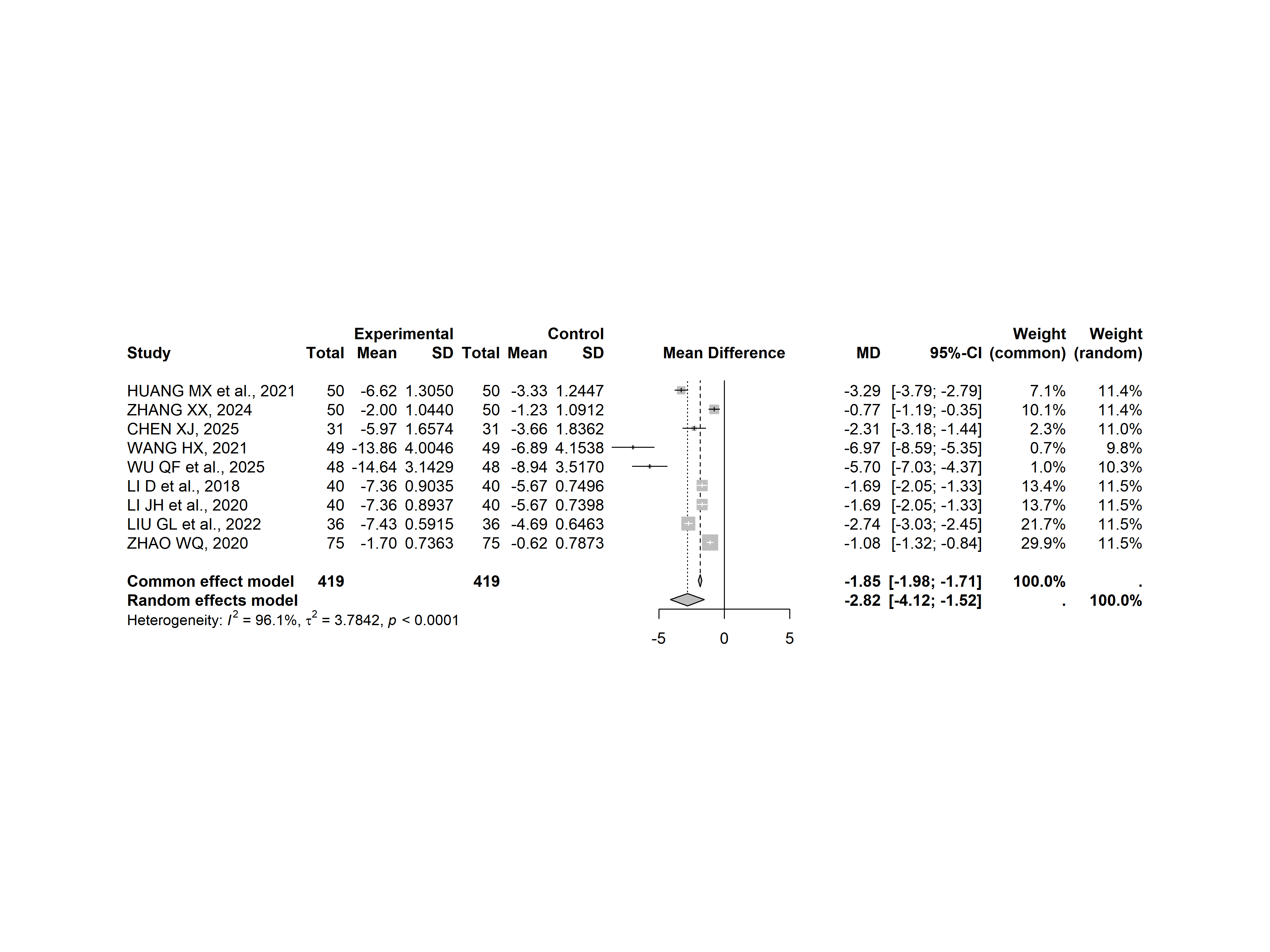

Supplement: Supplementary file 8 [file Image_8.PNG]

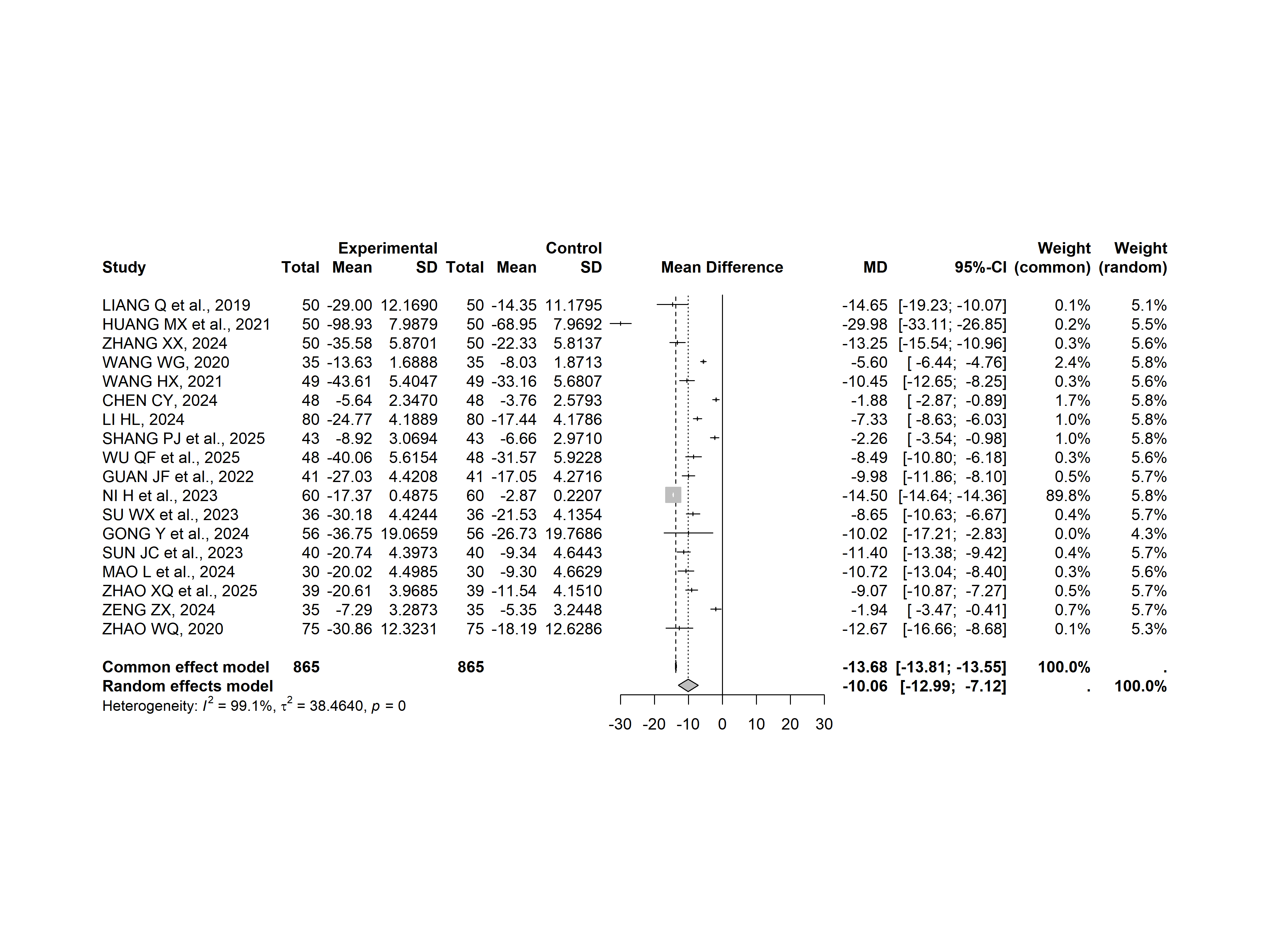

Supplement: Supplementary file 9 [file Image_9.PNG]

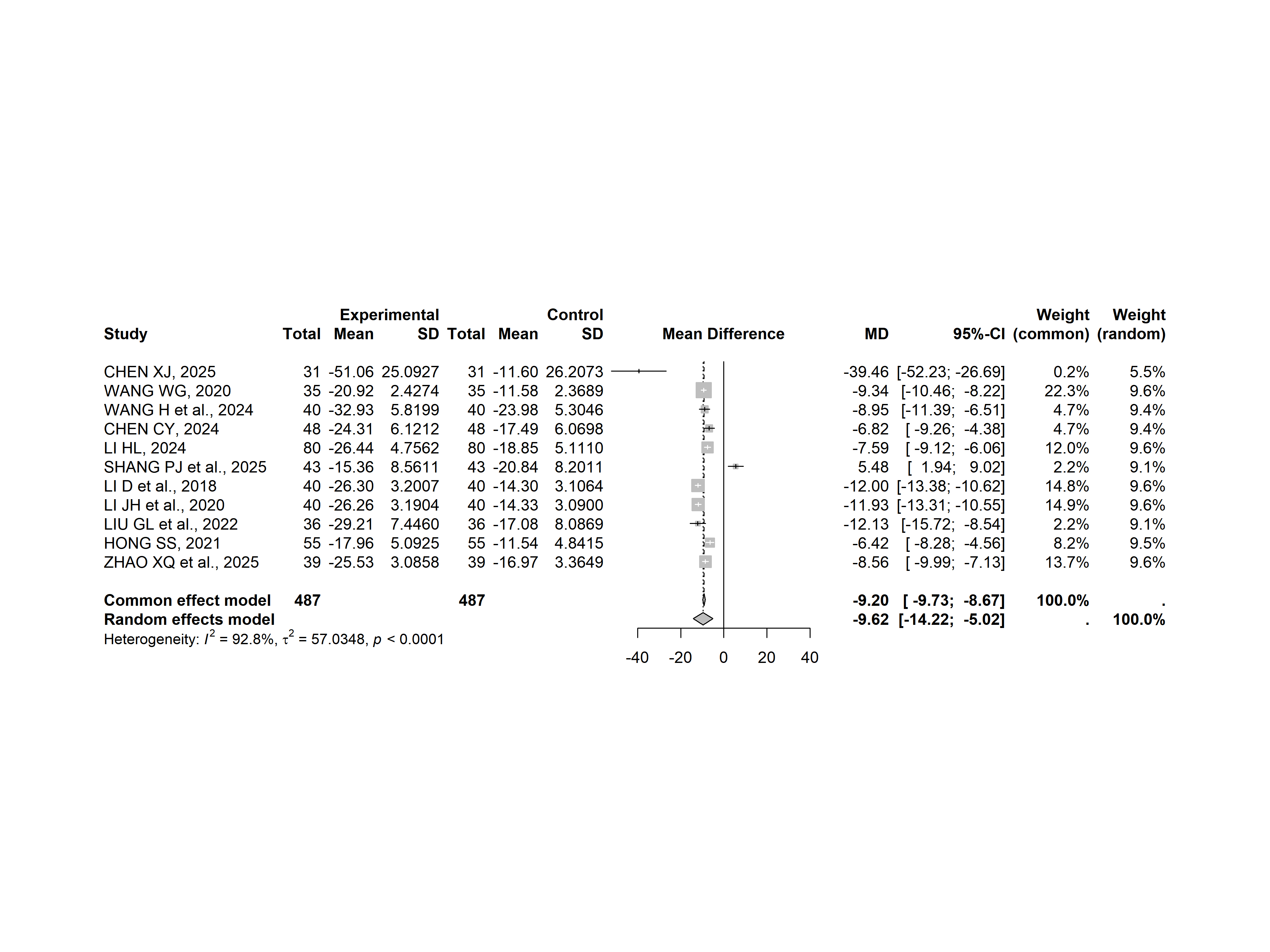

Supplement: Supplementary file 10 [file Image_10.PNG]

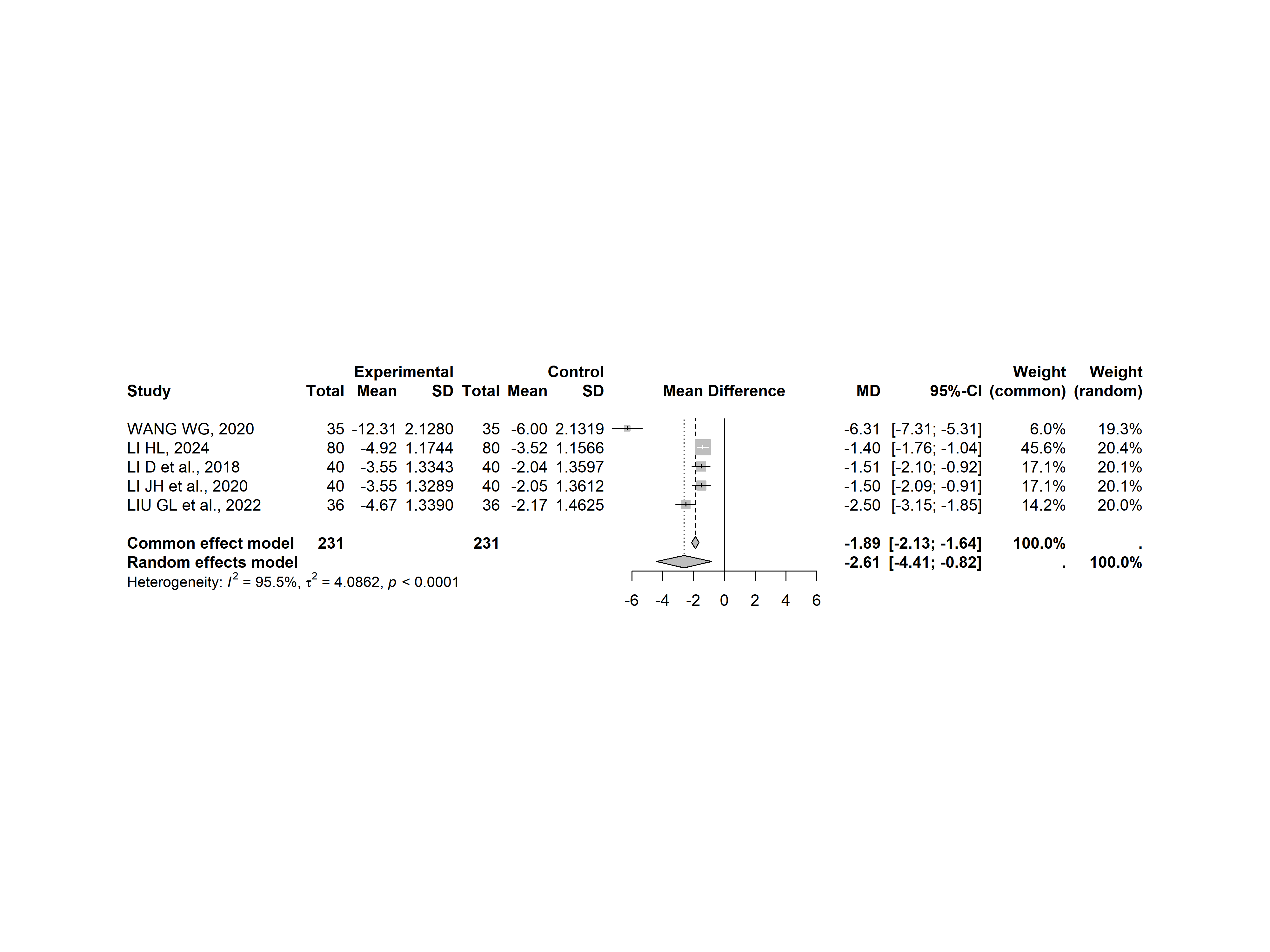

Supplement: Supplementary file 11 [file Image_11.PNG]

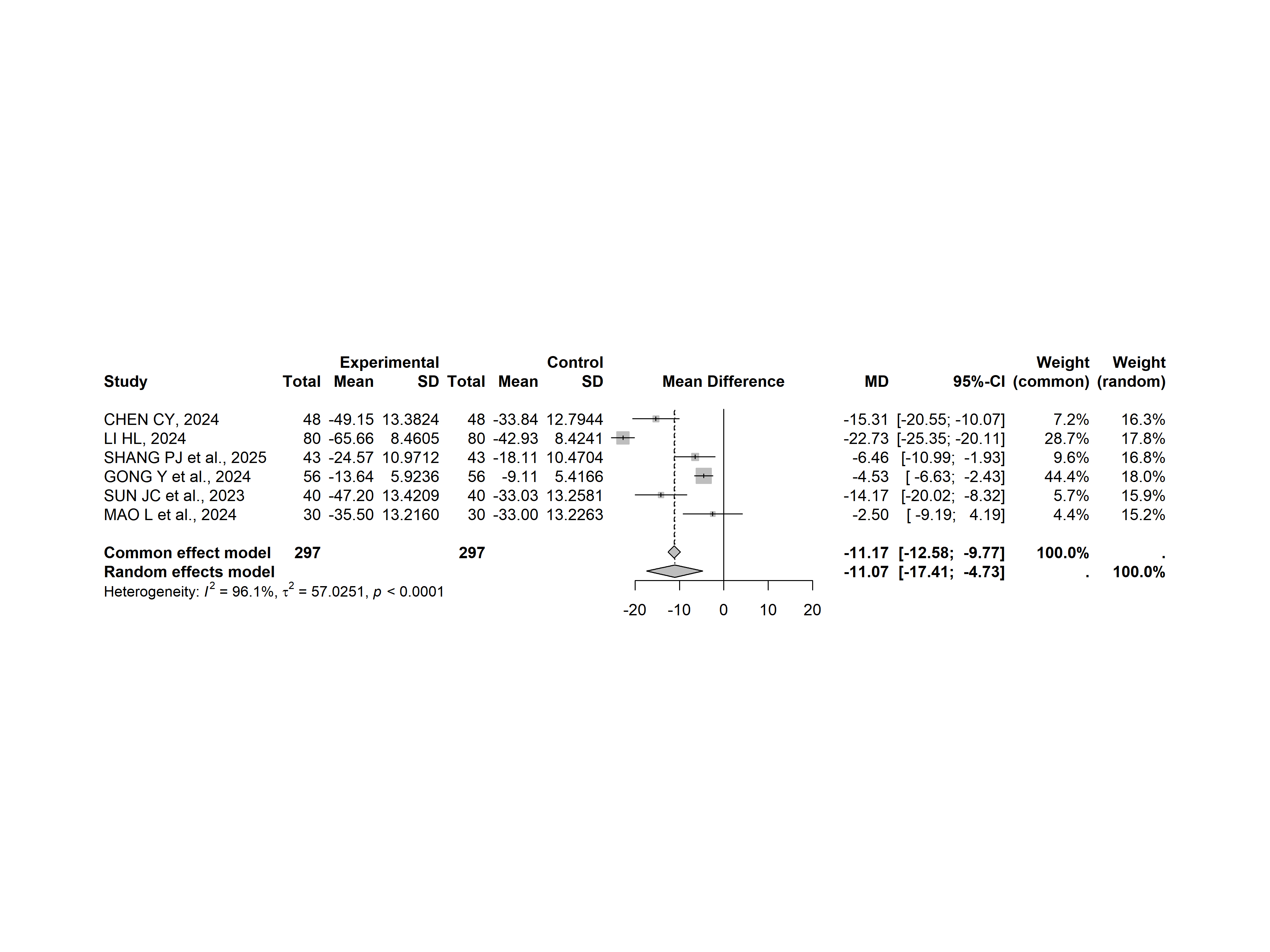

Supplement: Supplementary file 12 [file Image_12.PNG]
